# A two-stage inter-rater approach for enrichment testing of variants associated with multiple traits

**DOI:** 10.1038/ejhg.2016.171

**Published:** 2016-12-21

**Authors:** Jennifer L Asimit, Felicity Payne, Andrew P Morris, Heather J Cordell, Inês Barroso

**Affiliations:** 1 Wellcome Trust Sanger Institute, Hinxton, UK; 2 MRC Biostatistics Unit, Cambridge Institute of Public Health, Cambridge CB2 0SR, UK; 3 Department of Biostatistics, University of Liverpool, Liverpool, UK; 4 Institute of Genetic Medicine, Newcastle University, Newcastle upon Tyne, UK

## Abstract

Shared genetic aetiology may explain the co-occurrence of diseases in individuals more often than expected by chance. On identifying associated variants shared between two traits, one objective is to determine whether such overlap may be explained by specific genomic characteristics (eg, functional annotation). In clinical studies, inter-rater agreement approaches assess concordance among expert opinions on the presence/absence of a complex disease for each subject. We adapt a two-stage inter-rater agreement model to the genetic association setting to identify features predictive of overlap variants, while accounting for their marginal trait associations. The resulting corrected overlap and marginal enrichment test (COMET) also assesses enrichment at the individual trait level. Multiple categories may be tested simultaneously and the method is computationally efficient, not requiring permutations to assess significance. In an extensive simulation study, COMET identifies features predictive of enrichment with high power and has well-calibrated type I error. In contrast, testing for overlap with a single-trait enrichment test has inflated type I error. COMET is applied to three glycaemic traits using a set of functional annotation categories as predictors, followed by further analyses that focus on tissue-specific regulatory variants. The results support previous findings that regulatory variants in pancreatic islets are enriched for fasting glucose-associated variants, and give insight into differences/similarities between characteristics of variants associated with glycaemic traits. Also, despite regulatory variants in pancreatic islets being enriched for variants that are marginally associated with fasting glucose and fasting insulin, there is no enrichment of shared variants between the traits.

## Introduction

Apparent links between disease susceptibilities may be explained by shared genetic aetiology, such that a variant may be associated with multiple traits. Besides identifying shared associated variants, a further objective is to determine whether the overlap of associated variants between the traits may be related to SNP (or trait × SNP)-specific characteristics. Identification of specific characteristics that are predictive of overlap enables refinement of the set of variants in further searches for predisposing variants of both traits. Moreover, Bayesian priors may be defined such that a SNP belonging to a predictive category has a higher prior probability of association than SNPs outside that category; priors may also be allowed to differ so that the prior probability increases with the number of predictive categories that the SNP belongs to. The overall purpose of the proposed method, corrected overlap and marginal enrichment test (COMET), is to determine whether agreement (overlap) between the verdicts of association between a SNP and a phenotype can be related to SNP-specific (eg, functional annotation) or trait × SNP-specific characteristics, such as membership of known biological pathways.

Several existing methods address similar, but distinctive, objectives; for example, GoShifter,^1^ genetic analysis incorporating pleiotropy and annotation (GPA),^2^ and a method implemented in database for annotation, visualisation and integrated discovery (DAVID).^3^ All of these methods assess enrichment of annotations among trait-associated variants and, on application to shared variants between different traits, do not account for marginal enrichment of individual traits. Testing for annotation enrichment within trait-associated SNPs is the reverse of the proposed objective of testing for enrichment of trait-associated variants within annotations. In the latter, the number of associated variants is treated as the random variable, which aligns with the perception that we observe a number of associated variants and there are more to discover. In contrast, testing for annotation enrichment in a set of associated SNPs fixes the number of associations found and assesses annotation status among them; the annotation status is treated as the random variable in that approach.

With regards to overlap enrichment extensions, any of the single-trait enrichment methods may be extended by considering the set of SNPs associated with two traits. However, this does not automatically account for enrichment due to chance, as the marginal distributions of the individual traits are not accounted for. The GPA approach uses annotation information to increase the statistical power to identify risk variants. The authors of the method recommend caution in interpreting the enrichment testing approach of GPA with respect to overlap variants, as a significant *P*-value may be due to marginal enrichments.^2^ GoShifter uses a computationally intensive permutation approach^1^ and the test implemented in DAVID involves calculation of a hypergeometric probability.^3^ We apply DAVID to test for enrichment among shared variants, rather than variants associated with a single trait and demonstrate that it has an increased type I error rate. Owing to the inflated error, power comparisons are not carried out with DAVID.

COMET requires only summary statistics and is applicable to case–control or quantitative trait studies that may or may not have overlapping individuals. Simulations demonstrate that any degree of overlap between studies does not inflate the type I error for detection of SNP characteristics that are predictive of concordant associations between the traits. As COMET only requires fitting several linear models and does not depend on permutations to assess significance, it is computationally efficient. The data only needs to be clumped once, and then may be quickly analysed with any set of covariates. On a Linux (64 bit) machine with X86-64 architecture, 32 cores, and 2 × 2.1 Ghz 12 core AMD 6272 CPU, on data that has already been clumped, COMET is able to run for one pair of traits and one set of five covariates in 3 min, 44 s for our data application, where the fitting of the models takes 36 s.

There is flexibility in the covariates that may be incorporated in the analysis, leading to a range of potential applications. Before our real data application, we first examine the potential for a set of functional annotation covariates to differentiate between associated variants (with *P*<5 × 10^−6^, as given by the National Human Genome Research Institute (NHGRI) Genome-Wide Association Study (GWAS) catalogue^4^) for 14 different diseases/traits. COMET is then employed with these covariates to assess whether any annotation class is enriched for variants associated with fasting insulin, fasting glucose or 2-h glucose, or enriched for shared associations between any pair of the three glycaemic traits (from the Meta-Analyses of Glucose and Insulin-related traits Consortium; MAGIC). As more genome-wide significant loci have been identified for the glucose traits than for fasting insulin,^5^ an objective is to determine whether there are certain characteristics that are enriched for variants associated with either or both traits; such features may then be used for refinement of searches for further associated variants. On the basis of our results, we proceed with further analyses using COMET to test for enrichment of trait(s)-associated variants within tissue-specific regulatory regions. The software for COMET is freely available at http://www.sanger.ac.uk/science/tools/comet.

## Materials and methods

Studies of agreement are common in clinical studies and psychiatric research, where one is often interested in the agreement among expert/rater opinions. A special case is when the opinion/rating is a dichotomous outcome, such as a diagnosis. Inter-rater agreement approaches give a measure of the concordance between two raters (eg, physicians) that make a verdict or pronouncement (eg, disease presence/absence) on the same subject, and adjust for agreement between raters that may occur simply due to chance. A two-stage inter-rater agreement model identifies covariate categories containing more concordance/discordance in verdicts than expected by chance, accounting for the marginal rater opinions.^6^ We adapt this model to the genetic association setting to identify features predictive of shared associations at a SNP, accounting for the marginal trait associations; each ‘subject' corresponds to a SNP, whereas each ‘rater' corresponds to a trait. It may also be used to assess features predictive of association for individual traits.

At each genetic variant, a binary variable is defined for each trait corresponding to evidence of association with the trait, based on a pre-specified significance threshold; this corresponds to the verdict of each rater. Analogous to comparing measurements taken by raters on the same individuals, we compare measurements of trait-association at each SNP. Rather than considering agreement for both traits (ie, either having or not having association evidence at the same SNP), we focus only on both traits having association evidence, as lack of association evidence does not imply that the association does not exist (eg, due to lack of power).

Evidence of association for each trait with each SNP may be defined according to *P*-values or Bayes' factors (BFs). We focus on BFs, as BFs may be easily computed from summary statistics^7^ and have several advantages over *P*-values in the comparison of multiple studies.^8^ In both our simulations and data application, we used a Bayesian threshold of log_10_(ABF)>0.695 (based on threshold settings *R*=20, *π*_0_=0.99), corresponding to a *P*-value threshold of 0.004–0.01, depending on the study size;^8^ see Supplementary Information for BF details.

### Model

We consider SNP-specific and/or trait × SNP-specific covariates based on prior genetic information such as biological annotations. Covariate categories may then be tested for enrichment of (marginal and/or shared) associated variants. As the inter-rater methods assume independent subjects (with subjects here corresponding to SNPs), we first prune (*r*^2^>0.1) the set of SNPs (minor allele frequency (MAF) >5%) that comprise the GWAS data for each trait. The MAF threshold of 5% was chosen as we focus on GWAS results, though in application to other data sets (eg, large samples of exome data) lower MAF variants may be included. SNPs are clumped using *r*^2^>0.1 to satisfy the independence assumption required for the regression models. We make use of a joint association metric that accounts for the significance of a SNP with respect to each trait, maximising the retention of SNPs associated with multiple traits, rather than SNPs with high association evidence with one trait and not with the other^8^ (see Supplementary Information for details).

Let **x**_**i**_ be a vector of SNP-specific covariates, **x**_**ir**_ be a vector of SNP-trait-specific covariates, *Y*_ir_=1 (evidence of association at SNP *i* for trait *r*); *r*=1, 2, and *p*_ir_=Pr(*Y*_ir_=1|**x**_**i**_, **x**_**ir**_); *r*=1, 2; *i*=1,...,*m.* In the inter-rater model,^6^ agreement between the raters at subject *i* would be defined as *Y*_i_=*Y*_i1_*Y*_i2_+(1−*Y*_i1_) (1−*Y*_i2_). Instead, we focus on the concordance of associated SNPs, and therefore consider *Y*_i_=*Y*_i1_*Y*_i2_. The marginal models for conditional probability of a detected association given a particular trait (*r*=1, 2) are:


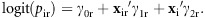


The intercept term *γ*_0r_ is the baseline probability of association, accounting for the probability of association that is not attributable to any of the covariates. An effect estimate that meets the significance threshold (eg, 0.05) and is positive suggests that SNPs within the coinciding covariate category tend to be associated with the trait (ie, positive enrichment); negative enrichment is present if the significant effect estimate is below zero. Collectively, this model tests for covariate categories that are predictive of SNP-trait associations.

These marginal models are first fit independently for each trait, then the fitted models are used to obtain estimates of the log-odds of chance overlap term 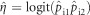, which accounts for chance overlap, assuming that the probabilities of association at each trait are independent (if modelling agreement rather than concordance of association one would instead have 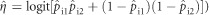). This term is then used as an offset term in the model for the probability of overlapping associations (or agreement):


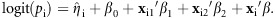


If overlap is due to chance alone, then all covariate effect estimates are not significantly different from zero and the probability of overlap is simply the product of the marginal probabilities, 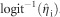. This observation helps us make inferences on the features of SNPs for which there is an enrichment of overlapping associations. A statistically significant intercept term *β*_0_ would be suggestive of more agreement than expected by chance that is not accounted for by any of the covariates. For instance, if SNPs associated with one trait tend to be associated with the other trait, but this sharing of associations is not related to any of the covariates, then the intercept term would account for this agreement. This framework may easily be extended to identify predictive features of shared SNPs for *R* traits by defining agreement at SNP *i* as 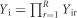. In our particular application to three glycaemic traits, there were only six SNPs that were shared between all three traits. Therefore, little inference could be made on the features of this small set of SNPs, and we proceeded by applying COMET to each pair of traits.

The traits may be from studies composed of disjoint sets of individuals or possibly from studies that share some individuals in common. In particular, for two quantitative traits, measurements for both traits may be taken on a portion of individuals. In the usual inter-rater set-up, different raters have correlated responses by the nature of rating the same subject, which is akin to correlation between trait associations expected in the presence of shared individuals, when testing at a certain SNP. This may influence the overall probability of concordance between the ratings but, intuitively, although this will affect the intercept term, this should not affect the tests of whether or not any of the covariates explain the concordance in the ratings. In the scenario of two case–control studies, there is the possibility of shared individuals between the control sets of the two studies. These shared controls may influence the individual SNP association tests, but by similar reasoning to the quantitative traits case, only the intercept term is expected to experience an impact. On a similar note, the traits may be correlated (eg, height and birth weight) or linked through a phenotypic derivation (eg, height and kg/m^2^), as the offset term accounts for each of the marginal distributions when testing for enrichment among shared variants.

Full marginal models for *p*_ir_ are recommended, such that any covariates that are considered for inclusion in the overlap model are included in each marginal model. This prevents spurious results in the overlap model for *p*_i_, as *p̂*_*ir*_ are needed to estimate the offset term.^6^ In the final overlap model, covariates of categories containing no overlap SNPS are removed.

**Figure 1:**
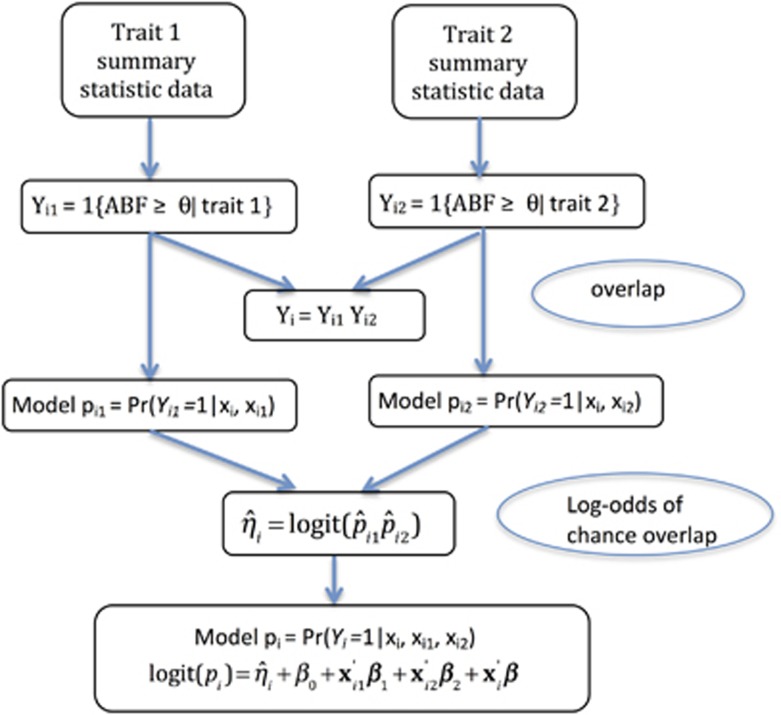
Flow chart of inter-rater approach to overlap analysis of two traits.

It has been noted that the variance estimates for each coefficient of the model for *p*_i_ assume that the offset term is known rather than estimated, so that alternative approximation techniques such as the jackknife are suggested.^6^ A jackknife estimate of the variance may be obtained by a leave-one-out procedure in which each subject (SNP) is removed and the two-stage models are fit to the data with one fewer subjects. However, as there are a large number of SNPs, there are negligible changes to the fitted models at the removal of each individual SNP. Therefore, for computational efficiency, we make use of the resulting coefficient estimates and standard errors from the model based on a known offset term. A flow chart for COMET is given in Figure 1.

### Covariates

Various SNP-specific covariates may be used to inform about overlap between traits, allowing flexibility in use of the method. A set of possible SNP-specific covariates is listed in Table 1, which is a modification of categories that have previously been considered when making use of prior knowledge for prioritising SNPs for follow-up.^9^ Covariate categories that each SNP is positive for are determined by the Variant Effect Predictor (VEP, v81) of Ensembl,^10^ which outputs all consequences of each variant on the protein sequence and gene expression, across all transcripts for the gene, so that a SNP may be positive for multiple covariate categories.

As a reference to the general features of SNPs, we examine the distribution of SNPs from the 1000 Genomes CEU samples, phase 3 release.^11^ On pruning the common SNPs (MAF>0.05) on *r*^2^*>*0.1 (using PLINK v1.07), there are 208 780 approximately independent variants. Table 1 provides the proportion of these SNPs that belong to each of the covariate categories, as well as the coinciding proportions for unpruned common SNPs. These proportions show a close correspondence, suggesting that the pruned SNPs reflect the overall distribution seen in the common SNPs in CEU of 1000 Genomes.

### Simulations

Each simulation is based on 208 780 approximately independent SNPs that remain after pruning the common SNPs on *r*^2^*>*0.1 in the 1000 Genomes CEU samples. Functional annotations for these SNP are obtained from VEP (v79). We focus on models that include five SNP-specific covariates that are listed in Table 1, namely Q1, Q2, Q3, Q5 and Q6 that are positive in 51.5%, 0.39%, 0.54%, 1.40%, and 64.1% of SNPs, respectively; Q4 is not included in the models as <0.025% of the pruned SNPs fall within this category. Several technical details regarding differences between these simulation proportions and those of Table 1 are detailed in the Supplementary Information.

For assessment of power, only one of the five covariate categories (Q1 or Q5) is set as enriched for overlapping associations between the traits, though this does not restrict causal SNPs from belonging to other categories. We consider various proportions *p*_12_' of variants that are associated with both traits and belong to the enriched category. The overall proportion of overlap variants is denoted by *p*_12_, whereas the marginal proportions of SNPs associated with traits 1 and 2 are given by *p*_1_ and *p*_2_, respectively. The simulation algorithm, parameter selection, and technical details are given in the Supplementary Information. For each parameter setting, we run 1000 replications to approximate type I errors and power. Type I errors are approximated from simulations that do not assign enrichment to any of the covariate categories, such that overlapping variants are present and there is no restriction on their allocation to covariate categories; this mimics the natural distribution of SNPs among the covariate categories. For further assessment of any inflation, we also consider QQ-plots of the standardised effect estimates compared with a standard normal distribution, as well as inflation factors (calculated from the median of *χ*^2^ distribution). As a comparison, type I errors for enrichment testing of overlap variants are also determined via the DAVID software.^3^

### Real data application

Before applying COMET to real data, we considered the distribution of the covariates among variants that are associated with fourteen traits/diseases. This pre-assessment illustrated that there is potential for the covariates to differentiate between trait-associated variants for different traits, as well as potential for identifying covariates that may be enriched for shared variants. Details and results on these comparisons are given in the Supplementary Information and in Supplementary Figure S5.

COMET was applied with the set of five functional annotation covariates to each pair of fasting insulin, fasting glucose and 2-h glucose, which were all measured on non-diabetic European-ancestry individuals (from MAGIC). The summary statistics from these glycaemic traits were downloaded from www.magicinvestigators.org and details on this dataset are provided in the Supplementary Information. Rather than restricting certain covariates to tests of positive enrichment (due to small covariate proportions) and others to two-sided tests (of positive or negative enrichment) in the overlap model, we simplify the presentation and focus only on positive enrichment. We further demonstrate how COMET could be used to explore regulatory annotation in greater depth by making use of an extensive database on regulatory information, RegulomeDB, which covers over 100 tissue and cell lines.^12^ In RegulomeDB, known and predicted regulatory DNA elements include regions of DNase hypersensitivity, binding sites of transcription factors, and promoter regions that have been characterised to regulation transcription.

Of particular interest are tissues that are involved in metabolism, i.e. pancreas, liver, cardiac muscle, skeletal muscle, and adipose tissues. Pancreatic islet cells are central in the pathogenesis of type 2 diabetes (T2D) and active islet enhancer clusters have been demonstrated to be enriched in T2D risk-associated and fasting glucose-associated variants.^13^ In addition, liver, adipose tissue, and skeletal and cardiac muscles develop insulin resistance as defence against damage from an excess nutrient load.^14^

Owing to the likely collinearity between the tissue-specific regulatory covariates, we ran separate models including one regulatory covariate annotated by RegulomeDB, for several filtrations on the tissue type(s); details of the specific cell/tissue lines within each tissue group are provided in the Supplementary Information. Initially, eight models were considered: one for each of the five metabolism-involved tissues, liver cancer (as a tissue that is involved in metabolism, but cancerous so may/may not be enriched for glycaemic trait-associated variants), the union of the five metabolism-involved tissues, and the collection of all tissues available in RegulomeDB. As the pancreatic tissue group consists of tissues from both pancreatic islets and the pancreatic duct, we also compared our results when only pancreatic islets are included. The respective proportions of pruned variants (*r*^2^<0.1) that are regulatory in each tissue type are 0.0768 (pancreas), 0.0666 (pancreatic islets only), 0.0779 (liver), 0.0275 (cardiac muscle), 0.116 (skeletal muscle), 0.0012 (adipose), and 0.0955 (liver cancer). On considering all (5) tissues involved in metabolism, the proportion is 0.166, or 0.162 if pancreatic duct tissues are excluded. Among all available tissues, the proportion of regulatory variants is 0.693.

## Results

### Simulation study

Two equal-sized case–control studies were generated, where study *r* (for trait *r*; *r*=1, 2) is composed of *N*_r_ cases and *N*_r_ controls; we consider study 1 with *N*_1_=3000 each of cases and controls and study 2 with *N*_2_=5000 each of cases and controls, as well as (*N*_1_, *N*_2_) taking values (5000, 10 000) and (10 000, 20 000). In our null simulations, the proportions of trait-associated variants for trait 1 (marginal), trait 2 (marginal) and shared between them are, respectively, *p*_1_=0.04, *p*_2_=0.02 and *p*_12_=5 × 10^−4^. For all five covariates, both sets of standardised effect estimates from the marginal models display a close alignment with the standard normal distribution (eg, see Supplementary Figure S1). The coinciding inflation factors for covariates Q1, Q2, Q3, Q5, and Q6 are, respectively, 1.07, 1.19, 1.09, 0.97, and 1.08, which are not substantially over-inflated, though the smallest category Q2 (containing <0.5% of the variants), appears to be most inflated.

For detecting positive enrichment of overlap variants at significance level *α*=0.05, type I error estimates for COMET and DAVID are given in Table 2. The type I errors of DAVID are consistently higher than those based on COMET, and the 95% confidence intervals for the three categories with fewer than 2% of the variants (Q2, Q3, Q5) are well above 0.05. COMET has a better controlled type I error rate, as the 95% confidence intervals contain 0.05 or have an upper bound that is slightly below it.

**Figure 2:**
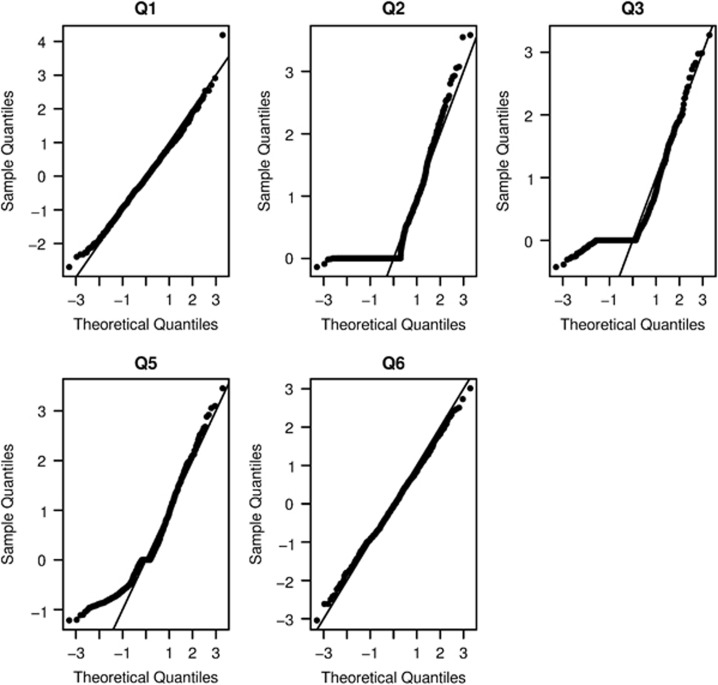
QQ-plots for the covariates in the most appropriate overlap model fit to simulated equal-sized case–control data (*N*_1_=3000 each and *N*_2_=5000 each). The model is fit to simulated data having *p*_1_=0.04, *p*_2_=0.02, *p*_12_=5 × 10^−4^ and no covariate categories are set-up as enriched.

Positive-enrichment overlap tests with COMET are well-calibrated for all covariates, though tests for negative enrichment are less well-calibrated for covariates Q2, Q3, and Q5 (eg, see Figure 2). As Q2, Q3, and Q5 harbour fewer than 2% of the variants, this proportion substantially decreases when we make the additional restriction that variants are detected as overlap variants. Consequently, approximately half of the simulations result in either an empty set of overlap variants in the covariate category, so that the covariate is excluded from the final overlap model, or a negative effect estimate that is not significantly different from 0; this behaviour is illustrated in the QQ-plots. The inflation factors for Q1 and Q6 are 0.83 and 0.93, while inflation factors calculated from the positive standardised statistics for Q2, Q3, and Q5 are 1.46, 0.62, and 1.05. In summary, one-sided tests for positive enrichment are well-calibrated for all covariates. There is inflation for Q2 and deflation for Q3, which, respectively, contain 0.39% and 0.54% of the variants, suggesting that the type I error rate is not controlled very well when fewer than 1% of the variants are positive for the covariate. In addition, two-sided tests for enrichment in either direction may be tested for in the larger categories, Q1 and Q6.

For assessment of power, we considered each of Q5 (1.4% of variants) and Q1 (51.5% of variants) as being enriched for overlap, so that any impact of the category proportions may also be assessed. Covariate categories that are not designed as enriched for overlap each give additional type I error results and can be averaged over the simulation settings for each covariate (Supplementary Table S1); individual results for all coefficients are given in Supplementary Tables S2 and S3. The average error rates shown in Supplementary Table S1 appear to have more stability than the individual rates.

**Figure 3:**
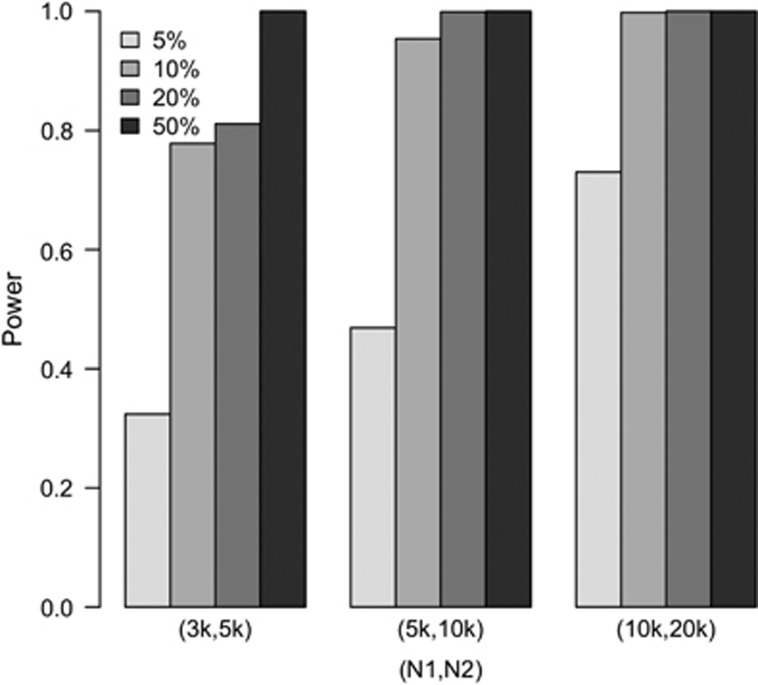
COMET power for detecting Q5 as a category positively enriched with overlap signals at coefficient significance level 0.05. In each of the 1000 simulations, the Q5 category (1.4% of common CEU SNPs LD-pruned at *r*^2^>0.1) was set to have a certain proportion of shared causal variants. The selected proportion of causal variants in this category *p*′_12_ is indicated in each column, followed by the proportion among the causal variants *p*′_12_/*p*_12_, as a percentage. Studies 1 and 2 are each equal-sized case–control studies of *N*_1_ each and *N*_2_ each, respectively. Type I error is denoted by bold font.

For power assessment, the proportion of overlap causal variants that fall within Q5 was assigned values from 5 to 50% (Figure 3; Supplementary Table S4). For (*N*_1_, *N*_2_) set at (5000, 10 000) or (10 000, 20 000), the detection power is close to 100% at 20% enrichment, and is high at 10% enrichment; high power near 80% is attained for (3000, 5000) when there is at least 10% enrichment. The enrichment setting of *p*′_12_=7 × 10^−6^ corresponds to the null hypothesis of no enrichment (see Supplementary Information for details), and the respective type I error estimates are 0.045, 0.039, and 0.035 for increasing study sizes. Results for Q1 in the case–control setting and all quantitative trait results are shown in the Supplementary Information.

### Application to glycaemic traits

Results of the positive enrichment tests from COMET applied to fasting glucose (FG), fasting insulin (FI) and 2-h glucose (2G) are given in Table 3. Among potentially deleterious SNPS (0.67% of pruned common variants), enrichment of overlap variants is detected for FG-2G (two variants) and for FI-2G (one variant); see Table 3.

In addition, SNPs in mature miRNAs that have a regulatory effect (ie, that are transcribed, though not translated) tend to be enriched for variants associated with each of the three glycaemic traits. Nonetheless, there are not more shared variants than expected by chance, considering these marginal enrichments; Our results also indicate that there is positive enrichment of variants associated with FG and with FG-2G among SNPs that overlap potentially regulatory or regulatory regions. Consequently, we tested tissue-specific regulatory annotations for positive enrichment in an additional analysis.

### Tissue-specific analysis of glycaemic traits

Results for tissue-specific analyses are shown in Table 4. Enrichment in adipose tissue is not detected, as it only contains 0.12% of the variants. Regulatory variants in pancreas tissues (and only pancreatic islets) are enriched for marginal associations with FG, FI, and 2G, as well as FG-2G shared variants, though they do not contain more FG-FI variants than would be expected by chance (Table 4). Analysis without accounting for the marginal distributions can be obtained by excluding the offset term, resulting in a reduction of the *P*-value to 0.044 (pancreas tissues), suggesting enrichment. This illustrates that marginal predictive factors are not necessarily predictive of overlap variants, with the offset term able to account for any perceived overlap that may in fact be due to chance. FI and FG associated variants are enriched in liver tissue regulatory variants, though 2G variants are not. COMET also detected that regulatory variants in cardiac muscle are enriched for FG and those in cardiac and skeletal muscle are each enriched for the FG-2G overlap.

Considering the five metabolic tissues collectively, there is enrichment of each individual trait, as well FG-2G, though these signals disappear when all available tissues are considered collectively. There is an absence of FI-FG enrichment signals in tissue-specific analyses and the collective tissue analysis suggests enrichment, but such overlap variants are regulatory in a range of tissues that may be contributing to the signal. The FI-FG SNPs (GRCh37/hg19 assembly) that are regulatory in at least one metabolism-involved tissue are listed in Supplementary Table S8, together with their nearest gene and associated phenotypes. In Supplementary Table S9, analogous information is given for the FI-FG overlap SNPs that are only regulatory in a tissue that is not involved in metabolism, such as tissues from cancerous liver, blood (cancerous and normal), cerebellum, skin, and bone marrow.

## Discussion

We have proposed COMET as a computationally efficient method that makes use of GWAS summary statistics to test categories for enrichment of variants that are associated with multiple traits, accounting for chance overlap due to the marginal associations of each trait; individual trait-specific tests of enrichment are also encompassed. In the association classification of variants we used a Bayesian threshold of log_10_(ABF)>0.695 (based on *R*=20, *π*_0_=0.99) that corresponds to a *P*-value threshold of 0.004–0.01, depending on the study size.^8^ This lenient threshold allows us to highlight new overlapping variants not already known to be genome-wide significant, and such variants that fall within an identified enrichment category (ie, a category predictive of overlapping association) may have a stronger prior probability for having true associations with each trait. Enrichment categories may also indicate a direction of refinement for future searches for overlap variants. For example, our analysis suggests that being a potentially deleterious variant is a predictive factor for shared associated variants between glycaemic traits. Therefore, further shared associations may be revealed through the analysis of whole-exome or whole-genome data, which are enriched for potentially deleterious variants that are generally poorly represented in other genome-wide association arrays.

As a means of pre-assessing the usefulness of a set of functional annotation covariates for our model, we compared the proportion of covariate-positive trait-associated variants (with *P*<5 × 10^−6^) for an assortment of traits. However, by considering the proportion of associated variants that are positive for each covariate there is a range of confidence interval sizes for the traits, as the confidence interval depends on the number of associated variants that are listed in the NHGRI-EBI GWAS catalogue.^15^ A further limitation is that the results in the GWAS catalogue rely on a variety of studies, having a range of sample sizes, which in turn influences the ability to detect trait associations within each study. Therefore, the ability to detect enrichment based on these proportions is heavily influenced by the number of listed trait-associated variants. This pre-assessment gives further support for our approach of detecting enrichment of associated variants within covariates, rather than detecting enrichment of covariates within associated variants.

In an application to glycaemic traits we detect enrichment of associated variants (marginal and/or shared) within several functional annotation classes, and identify well-established positive controls, together with their biological support. The two glucose traits appear to have more overlapping variants falling within some categories than expected by chance, suggesting that these two traits are similar to each other, as expected.

The missense variant rs1260326 (hg19 chr2:g.27730940T>C; in GCKR) is associated with all three traits, and genome-wide significant for FG,^16^ 2G,^17^ blood metabolite levels, cardiovascular disease risk factors, metabolic and lipid traits, gout, liver enzyme levels, and chronic kidney disease.^15^ Additional variants within *GCKR* are genome-wide significant for FI-related traits^16^ and Crohn's disease. An additional missense variant rs13266634 (hg19 chr8:g.117172544C>T; in *SLC30A8*) is associated with both FG and 2G, and genome-wide significant for T2D,^18^ FG,^16^ fasting proinsulin levels,^19^ and glycated haemoglobin levels.^20^ These results are positive controls, since the variants were known to be genome-wide significant for the traits and our method both detects this overlap and suggests that these numbers are greater than expected by chance.

The gene *TCF7L2* is known to be associated with T2D and glycaemic traits^13^ and within it we identify two overlap SNPs that are in low LD (*r*^2^=0.089) with each other: rs7903146 is detected for each pair of traits and rs7079711 is identified for FI-FG. The SNP rs7903146 acts as a positive control, since it is the lead SNP in *TCF7L2* for associations with T2D,^18^ FI-related traits (interaction with BMI),^5^ and FG-related traits (interaction with BMI)^5^ and is also genome-wide significant for 2G^16^ and FG;^16^ this SNP is also our top signal for the FI-2G overlap and for each of FG and 2G.

A further positive control is detection of the FG-2G variant rs11708067 (in *ADCY5*), which is known to be associated with FG^16^ and is in LD with a known 2G-associated SNP rs2877716 (*r*^2^=0.807).^17^ Each FI-2G variant that is regulatory in a metabolism-involved tissue is within a gene containing FI- or T2D-associated variants (*P*<5 × 10^−6^).

The top FI-FG signal is rs6984305 (in RP11-115J16.1), which is regulatory in tissues from the pancreas, liver, cardiac muscle and skeletal muscle. In the MAGIC data under analysis, this SNP is genome-wide significant for FG (*P*-value 2.67 × 10^−8^; ABF 5.63) and highly significant for FI (*P*-value 3.36 × 10^−7^; ABF 4.10); rs6984305 is also in LD (*r*^2^=0.614) with a known genome-wide significant FG (interaction with BMI)-associated SNP, rs4841132.^5^

Several SNPs are of interest for further investigation, as they (and SNPs in LD with them) have not been previously identified as associated with glycaemic traits. The SNP rs4736324 (in LYPD2, which harbours variants associated with body fat distribution) is regulated in pancreas tissue/islets and is a FG-FI variant. Likewise, rs2014712 (in KCNK9 and regulated in liver tissue) is an FG-FI variant and variants in KCNK9 are associated with adiponectin levels, cholesterol and CAD. Variant rs598725 (downstream RP4-60717.1) is a FG-2G variant and is regulatory in both skeletal and cardiac muscles. Most of the overlap SNPs that are regulatory in a non-metabolic-involved tissue are not in LD with a variant that is associated (at *R*=20, *π*_0_=0.99) with more than one glycaemic trait. The exception is rs17036328 (within PPARG), which is in perfect LD with several variants that meet significance for each of FG, FI (genome-wide level) and 2G; two of these perfect LD variants are regulatory in cardiac and skeletal muscles.

Enrichment of variants associated with FG, FI, 2G, and FG-2G among regulatory variants in pancreatic islets concurs with the result that islets are enriched in loci that are associated with FG and T2D.^13^ Among regulatory variants in liver tissue, there is enrichment of FI and FG variants, though not 2G variants, aligning with the finding that individuals with impaired FG have hepatic insulin resistance, while those with impaired glucose tolerance (as measured by 2G) have normal to slightly reduced hepatic insulin sensitivity.^21^ This suggests that the liver plays a relatively more important role in influencing FG than 2G. Enrichment of FI-associated variants in liver tissue may coincide with insulin regulating glucose production in the liver during the fasting state. Enrichment of glucose trait variants in cardiac and skeletal muscle is likely linked with muscle being a target organ for insulin.

A possible limitation of the proposed approach is that the SNPs included in the analysis need to appear in both trait data sets, though imputed results are often available, so this may not have a significant impact. It is possible that, as we are limited by the set of SNPs available in both studies, the associated SNP may be a tag SNP for the causal variant, which is in a different covariate category, so that the enrichment category does not contain this causal variant. However, for covariate categories with a proportion of SNPs >1%, there would need to be some number of associated variants within the category in order for enrichment to be detected. It is highly unlikely that the majority of associated SNPs in the detected enrichment category are each a tag SNP for a causal variant in a different category. Therefore, even if this is true for an associated SNP, there is no change to the general biological interpretation of the covariate category being enriched for associated SNPs, as a set of associated SNPs has been detected in the category.

Alternative covariates to functional annotations may be trait × SNP-specific, to inform about whether overlap SNPs occur more likely than by chance within a certain trait feature, such as previously identified trait-associated SNPs (using information obtained from NHGRI-EBI). Additional covariate possibilities include SNP presence/absence in at least one gene (+/−50 kb buffer region) that has been identified as harbouring a trait-associated variant (*P*<5 × 10^−8^), or a less stringent classification (5 × 10^−4^<*P*<5 × 10^−8^), to increase the chances of finding novel results.

The proposed approach may also be used for pathway-based analyses, where the covariate indicates whether or not the SNP is in a certain pathway, of relevance to one of the traits. For genes in a given pathway (or group of related pathways), a covariate may be defined according to presence/absence of the variant within at least one gene (+/−500 kb buffer) in the pathway; an additional covariate may be defined as presence/absence of variant 500 kb away from gene and closer than 1000 kb. This pair of covariates may be used in a separate overlap model for each pathway (or pathway group) of interest.

In conclusion, our proposed procedure for identifying features predictive of overlap informs biological interpretation and enables refinement of the set of variants considered in further searches for predisposing variants for both traits.

## Figures and Tables

**Table 1:** Details of possible SNP-specific binary covariates and their distribution among common SNPs in the 1000 Genomes CEU samples

*Covariate*	*Description*	*Details*	*CEU (unpruned)*	*CEU (pruned)*
Q1	SNP in a transcribed but not translated region	Mature miRNA variant; non-coding transcript exon variant; intron variant; NMD transcript variant; non-coding transcript variant	0.513	0.517
Q2	SNP in a translated region but does not change the amino acid	Stop retained variant; synonymous variant; incomplete terminal codon variant;	0.00334	0.00380
Q3	SNP is potentially deleterious	Inframe insertion; inframe deletion; missense variant; initiator codon variant; splice region variant; stop lost	0.00341	0.00422
Q4	SNP is potentially loss of function	Stop gained; frameshift variant; splice donor variant; splice acceptor variant; start lost;	0.000104	0.000139
Q5	SNP is in a potentially regulatory or regulatory region	5′ UTR variant; 3′ UTR variant; TF-binding site variant; regulatory region ablation; regulatory region amplification; regulatory region variant	0.135	0.144
Q6	SNP is intergenic	Intergenic variant; upstream gene variant; downstream gene variant	0.635	0.638

The proportion of common MAF CEU 1KG SNPs that are positive for the covariate is given by CEU unpruned, whereas the coinciding results for SNPs pruned at *r*^2^>0.1 is given by CEU pruned. Results are based on VEP v81.

**Table 2:** Estimates of type I error (including 95% confidence intervals) for the detection as a category positively enriched with overlap signals at coefficient significance level 0.05, for the null enrichment setting with equal-sized cases and controls

	*Type I error (*H_*1*_: β*>0) for each covariate*									
	*Q1*		*Q2*		*Q3*		*Q5*		*Q6*	
N_*1*_ N_*2*_	*COMET*	*DAVID*	*COMET*	*DAVID*	*COMET*	*DAVID*	*COMET*	*DAVID*	*COMET*	*DAVID*
3000 5000	0.032 (0.021, 0.043)	0.059 (0.044,0.074)	0.061 (0.046, 0.076)	0.096 (0.078,0.114)	0.046 (0.033, 0.059)	0.142 (0.120, 0.163)	0.063 (0.048, 0.079)	0.114 (0.094, 0.134)	0.038 (0.026, 0.050)	0.061 (0.046, 0.076)
5000 10 000	0.048 (0.037, 0.061)	0.056 (0.042, 0.070)	0.058 (0.044, 0.072)	0.115 (0.095, 0.135)	0.044 (0.031, 0.057)	0.110 (0.091, 0.129)	0.052 (0.038, 0.066)	0.074 (0.058, 0.090)	0.048 (0.035, 0.061)	0.061 (0.046, 0.076)
10 000 20 000	0.053 (0.039, 0.067)	0.061 (0.046, 0.076)	0.058 (0.044, 0.072)	0.103 (0.084, 0.122)	0.049 (0.036, 0.062)	0.113 (0.093, 0.133)	0.044 (0.031, 0.057)	0.078 (0.061, 0.095)	0.048 (0.035, 0.061)	0.071 (0.055, 0.087)

Study *r* has *N_r_* each of cases and controls, *r*=1, 2.

**Table 3:** Results of the marginal and pair-wise inter-rater models overlap models fit to fasting glucose, fasting insulin and 2-h glucose

		*Covariates*					
*Trait models*	*Quantity*	*Q1: transcribed, not translated*	*Q2: translated, no amino acid change*	*Q3: potentially deleterious*	*Q5: potentially regulatory or regulatory*	*Q6: intergenic*	*Intercept: related to baseline association probability for marginal and shared beyond chance*
Fasting insulin (FI)	*P*-value Estimate STD error Count	**0.0326** **0.141** **0.0766** **766**	0.844 −0.466 0.450 5	0.189 0.258 0.293 12	0.113 0.0833 0.0687 271	0.194 0.0673 0.0778 836	**<2 × 10**^−**16**^ **4.35** **0.0873**
Fasting glucose (FG)	*P*-value Estimate STD error Count	**2.39 × 10**^−**4**^ **0.246** **0.0704** **920**	0.974 −1.13 0.5790 3	0.491 0.00719 0.305 11	**0.0494** **0.105** **0.0633** **322**	0.064 0.108 0.0709 960	<**2 × 10**^−**16**^ −**4.27** **0.0804**
2-h glucose (2G)	*P*-value Estimate STD error Count	**0.0416** **0.190** **0.110** **353**	0.624 −0.183 0.580 3	0.741 −0.376 0.580 3	0.356 0.0375 0.102 122	0.0675 0.168 0.112 399	<**2 × 10**^−**16**^ −**5.20** **0.127**
(FG, FI)	*P*-value Estimate STD error Count	0.308 0.183 0.364 29	1 0 NA 0	0.216 0.799 1.01 1	0.623 −0.112 0.357 10	0.167 0.361 0.374 32	0.339 0.414 0.433
(FG, 2G)	*P*-value Estimate STD error Count	0.544 −0.0637 0.578 15	1 0 NA 0	**3.98 × 10**^−**5**^ **2.94** **0.745** **2**	**0.0337** **0.928** **0.437** **9**	0.832 −0.517 0.537 11	0.302 0.661 0.640
(FI, 2G)	*P*-value Estimate STD error Count	0.305 0.327 0.639 9	1 0 NA 0	**0.0102** **2.43** **1.046** **1**	0.783 −0.598 0.763 2	0.265 0.415 0.659 10	0.946 0.0516 0.763

Tests of positive enrichment are performed for all covariates and bold font indicates significance at level 0.05. Cell values of (1, 0, NA) indicate that the covariate was excluded from the final overlap model. Two-sided *P*-values are given for intercept estimates.

**Table 4:** *P*-values for the positive enrichment of glycaemic trait-associated variants (at Bayesian decision criteria *R*=20, *π*_0_=0.99) in six different tissues, as well as all tissues available in RegulomeDB

*Trait/tissue*	*Pancreas (pancreatic islets only)*	*Liver*	*Cardiac muscle*	*Skeletal muscle*	*Adipose*	*Liver cancer*	*All (5) tissues involved in metabolism (panislets, rather than pancreas*	*All tissues*
Fasting insulin (FI)	**0.00779** (**0.00472**)	**0.0158**	0.294	0.183	0.687	0.390	**0.00189** (**0.00274**)	0.401
Fasting glucose (FG)	**0.00590** (**0.0116**)	**0.0200**	**0.00664**	0.139	0.739	**0.0453**	**0.00776** (**0.00670**)	0.0584
2-h glucose (2G)	**0.000556** (**0.00210**)	0.140	0.910	0.071	0.389	0.604	**0.0420** (**0.0488**)	0.0900
FI and FG	0.263 (0.153)	0.733	0.237	0.587	1.000	0.393	0.223 (0.416)	**0.0317**
FI and 2G	0.492 (0.418)	0.691	0.506	1.000	1.000	0.643	0.433 (0.552)	0.680
FG and 2G	**0.0327** (**0.0112**)	0.315	**0.0366**	**0.0480**	1.000	0.582	**0.00429** (**0.0129**)	0.344

Pancreas tissue includes tissues from both pancreatic ducts and pancreatic islets. Bold values indicate significance at level 0.05.
