# Supplementary material for: A two-stage inter-rater approach for enrichment testing of variants associated with multiple traits
Source: Eur J Hum Genet. 2016 Dec 21;25(3):341–9. doi: 10.1038/ejhg.2016.171 (PMC5302181; doi:10.1038/ejhg.2016.171)
Supplement: Supplementary Information [file ejhg2016171x1.docx]

**Supplementary Information**

**S.1. Approximate Bayes’ factors, Threshold Selection, and a Joint Metric**

Based on summary genetic association data from a regression (estimates of $\hat{\beta}$ and *V* = Var($\hat{\beta}$)), for each trait an approximate Bayes’ factor (ABF) may be calculated at each variant:

$$ABF=\sqrt{\frac{V}{V+W}}\exp\left(\frac{W}{V+W}\frac{Z^{2}}{2}\right)$$

where $Z=\hat{\beta}/\sqrt{V}$, $\hat{\beta}\sim N(\beta,V)$, $\beta\sim N(0,W)$ and $N(\mu,\sigma^{2})$denotes that the random variable follows a Normal distribution with mean $\mu$ and variance $\sigma^{2}$ (ref 1). In the case-control setting, a widely used default value for *W* the prior variance of $\beta=\log(OR)$ in an additive model is W=0.04 (ref 2). For standard Normal or inverse-rank normalised quantitative traits W=0.02 may be used, while an approximation for non-unit variance quantitative traits is W=0.02×Var(trait).

A threshold *θ*  for testing the null hypothesis of no association at a SNP is given by *θ* = PO/R, where PO=π_0_/(1-π_0_) is the prior odds of no association, π_0_ is the prior probability that there is no association at the SNP, and R = type II error cost/type I error cost. A SNP is considered to have evidence of association with a trait if ABF > *θ*. Genome-wide significance is usually set as log_10_*θ * between 4 and 6, based on *R*=1 and 1-π_0_ set to 10^-4^ – 10^-6^ (ref 3). The level of discovery increases as *R* rises above 1, since the cost of failing to identify an associated variant is higher than the cost of falsely detecting a null associated variant. In overlap analyses a less stringent threshold might be considered, so that a discovery setting is favoured for detection of associations in both traits, which can later be validated in replication studies. For this reason, we focus on *R=20* and π_0_ = 0.99 in our analyses, which coincides with association evidence at a SNP when log_10_(ABF) > 0.695. As a reference, for case-control studies this coincides with approximate marginal type I errors of 9.94×10^-3^ for equal-sized case-control samples of N=3000 each and 4.15×10^-3^ when N=20 000 (Ref 4). A more lenient threshold will identify a larger number of variants marginally associated with each trait and, apparently, more shared variants, though these may not necessarily show genuine evidence of overlap, once the marginal distributions have been accounted for.

For pruning based on two traits, we consider a joint association metric that has been defined for ABFs and for p-values^4^. At a given SNP, let ABF_1_ and ABF_2_ be the respective ABFs for traits 1 and 2, and let M be the maximum ABF observed at any SNP, for either trait. An association metric for pruning may then be defined by

ABF^*^ = max(ABF_1_, ABF_2_) + M×I(ABF_1_ > PO/R and ABF_2_ > PO/R),

where I(E) is the indicator function, taking on value 1 when event E = {ABF_1_ > PO/R and ABF_2_ > PO/R} holds and 0, otherwise^4^. SNPs are then ordered by decreasing ABF^*^ and a SNP is pruned out if it is in LD (r^2^ > 0.1) with a SNP having larger ABF^*^ and within 500 kb; this is implemented via the clumping algorithm in PLINK v1.07 (Ref 5) by assessing association evidence based on –log_10_ABF^*^ rather than p-values. A similar approach may be used for p-values^4^.

**S.2 Simulation Study Details**

There is a difference in the versions of VEP^6^ used for the simulations and for the data analysis; VEP v79 was used to conduct the simulations and, due to technical updates, was no longer accessible at the time of data analysis, so that VEP v81 was used in data applications. However, there is very little difference in the proportions that result from the two versions.

There is flexibility in the selection of covariates for the inter-rater approach, and for the purpose of demonstrating the method via simulations, we do not specify variants in regulatory regions, as described by the Ensembl Regulatory build (<http://www.ensembl.org/info/genome/funcgen/index.html>). Although, the Ensembl Regulatory build is employed in the data analysis, its lack of use in the simulations only results in a different covariate distribution for Q5 and does not impact any analyses. On the contrary, as Q5 is 1.4% (as opposed to near 14%, as when the Ensembl Regulatory build is called) in the simulations, this allows assessment of a wider range of covariate distributions; we are able to show that the type I error rate of COMET is well-controlled for testing positive enrichment when the covariate has a null distribution near 1%, and is not controlled as well when fewer than 1% of the variants are positive for the covariate.

Causal SNPs are selected to satisfy the proportion parameters *p_1_, p_2_, p_12_, p’_12_* in a nested manner. That is, for the *m* SNPs in the simulation, causal variants are selected in the following steps, where $\left\lceil u \right\rceil$: denotes the smallest integer larger than u (i.e. ceiling of *u*).

1. randomly choose $\left\lceil p_{12}^{'} \times m \right\rceil$ shared causal SNPs within enriched category
2. randomly choose $\left\lceil{(p_{12}-p}_{12}^{'}) \times m \right\rceil$ shared causal SNPs outside enriched category
3. randomly choose $\left\lceil\left( p_{1}-p_{12} \right) \times m \right\rceil$ trait 1 -specific causal SNPs from remaining unselected SNPs
4. randomly choose $\left\lceil\left( p_{2}-p_{12} \right) \times m \right\rceil$ trait 2 -specific causal SNPs from remaining unselected SNPs

Under the null hypothesis of no enrichment in covariate category C, the proportion of shared causal SNPs within that category is expected to be , where $p_{C}$ denotes the proportion of SNPs in the covariate category to be tested for enrichment; under H_0_, $p_{12}^{'}=p_{12}\times p_{C}$. It follows that there is positive enrichment of shared causal SNPs in covariate category C when $p_{12}^{'}>p_{12}\times p_{C}$ , which is indicative of more shared variants in category C than expected by chance; this is detected if the corresponding overlap model coefficient is both positive and significant at level α. In contrast, if this inequality is in the opposite direction, the category contains fewer shared variants than expected by chance and has negative enrichment. Likewise, such tests may be performed for the marginal models, where the proportion of causal variants for trait k that are contained with category C is $p_{k}\times p_{C}$ under the null hypothesis.

**Case-control Settings**

It is not expected that the marginal proportions of association with each trait will influence the results, as our objective is to identify the characteristics of shared associated variants between the traits. For case-control simulations, we fix *p_1_* = .04, *p_2_* = .02 and *p_12_* = 5×10^-4^, while varying *p’_12_* over a range of proportions of overlap SNPs in the enriched category; *p_12_* was selected based on previous overlap association results at *R=20* and π_0_ = 0.99 (Ref 4). The ORs at causal variants for each trait are uniform random variables between 1.08 and 1.2, and independently generated for each trait.

For covariate Q1 we have *p_C_* = 0.515, so that if *p_12_ =* 5×10^-4^, then under the assumption that H_0_ holds, we have *p’_12_* = 2.575×10^-4^. Therefore, for the case-control simulations with Q1 as the enrichment category we consider p’_12_ = 2.6×10^-4^, as well as p_12_ values of 1×10^-4^ and
4×10^-4^, corresponding to 20% and 80% of the shared causal variants, respectively. Positive enrichment of 80% falls within the confidence interval for fasting glucose associated variants (see Results section on Covariate Distributions), whereas negative enrichment of 20% serves for illustration of the method. In contrast, as Q5 has *p_C_* =0.014, under H_0_ we have *p’_12_ =* 7×10^-6^*.* This indicates that only tests of positive enrichment for shared variants may be considered for this covariate. Therefore, we consider the null setting of 1.4% of the causal variants being shared between the two traits, as well as overlap enrichment proportions coinciding with 5%, 10%, 20% and 50%. Further support of testing only for positive enrichment is given by our simulation results.

**Quantitative Trait Settings**

In our quantitative trait simulations, each trait follows a Normal distribution and we allow each causal variant to contribute approximately 0.1% of the phenotypic variance for each trait. We select p_12_= 0.0002 based on the observed overlap association results detected (*R=20* and π_0_ = 0.99) in glycaemic traits (MAGIC) and allow p’_12_ to vary. Therefore, among the 208,780 variants, 42 variants are selected as associated with both traits and the cumulative contribution to phenotypic variance for each trait is approximately 4%.

For each trait, the causal variants are independently assigned effects between 0.01 and 0.1 (odds ratios between 1.01 and 1.11), which align with the effect sizes detected for glycaemic traits (MAGIC) to date^7-9^. As p_12_ = 2×10^-4^, it follows that p’_12_=1×10^-4^ under the null hypothesis for Q1, whilst p’_12_=2.8×10^-6^ under H_0_ for Q5. Under the same rationale described in the case- control settings, in the overlap model, only tests of positive enrichment may be considered for covariate Q5.

Here, we consider 15,000 individuals in study 1 and 50 000 individuals in study 2, along the lines of the sizes used for glycaemic traits in the Meta-Analyses of Glucose and Insulin-related traits Consortium (MAGIC)^7,8^; there are approximately 15 000 individuals with 2-hour glucose measurements, while each of the fasting insulin and fasting glucose measurements are available for approximately 50 000 subjects. As with the case-control setting, both pairs of independent studies and studies with overlapping subjects are considered; we consider an overlap of 10 000 subjects between a study of size 15 000 and a study of size 50 000. We also consider larger proportions of shared individuals, where both studies consist of 11 000 individuals each, of which 10 000 (e.g. 91%) are shared between them, as well as the setting where both studies are composed of the same set of 10 000 individuals (all samples are shared) and settings where one study of 10 000 individuals is contained within the larger study of 12 000 (83% overlap for study 2) or 15 000 (67% overlap for study 2) individuals; see Supplementary Table S6.

**S.3 Glycaemic data details**

The insulin summary statistics are from a meta-analysis of 26 studies in up to 51 750 individuals^7,8^, while fasting glucose results are from an analysis of 29 studies in up to 58 074 subjects^7^ and 2-hour fasting glucose results are from an analysis of nine GWAS in 15 234 participants^8^. Fasting insulin values have been naturally log transformed, while both fasting glucose (mmol/l) and 2-hour glucose are untransformed, and the resulting summary statistics are based on regression models that adjust for BMI, age and sex, as well as study-specific adjustments^7,8^.

The LiftOver tool [<http://genome.sph.umich.edu/wiki/LiftOver>] was used in order to bring the MAGIC data to build 37. There were 2 121 488 SNPs present for all three traits that had MAF > 0.05 in the 1000 Genomes CEU population reference panel, and 93 811 SNPs remained after LD clumping (*r^2^>* 0.1) based on the multi-trait association metric described in the Methods. The distributions of these SNPs across the five covariate categories explored in the simulations are similar to that of Table 1 for the CEU population of 1000 Genomes. In particular, the proportions for Q1, Q2, Q3, Q5 and Q6 are 0.534, 0.0056, 0.0067, 0.183 and 0.618 in the clumped data. The coinciding proportions in the unclumped data are 0.513, 0.0052, 0.0059, 0.183, and 0.617, indicating that the clumped SNPs reflect the distribution of the entire set of common SNPs.

**S.4 Simulations Results**

**Case-Control**

We consider the common setting where there may be a large number of control samples that are available to both studies, resulting in an overlap of control samples. We examine the type I error rate when study 1 has 5000 cases, study 2 has 10 000 cases and both have 10 000 controls each, where among the 10 000 controls, 9000 are shared and each has an additional 1000 that are distinct (i.e. 90% overlap in controls). Similar results are obtained to the non-overlapping controls setting; type I errors are given in Table S1 and the coinciding QQ-plots are given in Figure S2. Inflation factors for Q1 and Q6 are 0.95 and 0.93, while inflation factors calculated from the positive standardised statistics for Q2, Q3, and Q5 are 0.75, 0.81, and 1.09. Also, the same issue mentioned above regarding poor calibration for tests of negative enrichment for categories Q2, Q3, and Q5 is again observed.

We next consider Q1 and test for positive enrichment, as well as a test of enrichment in either direction. There is a clear symmetry in the two-sided tests of enrichment, as the power to detect enrichment when 20% of the causal variants overlap between the two traits is of similar magnitude to when the percentage is 80% (see Table S3). The impact of sample size is more pronounced for the larger category Q1, compared to Q5, though the same degree of detection is not attained for Q1. When Q1 contains 80% of the overlap variants the power to identify it as an enrichment category for overlap increases by 20% with each increase in sample size that we considered (Table S3). Detailed results for all coefficients are given in Table S5.

Quantitative traits

The quantitative trait setting displays similar performance to that of case-control. For marginal models, the type I error for two-sided tests of enrichment are well-calibrated in the same manner as for case-control; QQ-plots are not provided as they are very similar to Figure S1 for the case-control setting. For instance, a sample of size 15 000 has inflation factors of 0.919, 0.896, 1.041, 0.982, and 0.957 for covariates Q1, Q2, Q3, Q5, and Q6.

Complete type I error results (at level 0.05) for the overlap model are given in Table S6 for study 1 consisting of 15 000 subjects, study 2 containing 50 000 individuals and either there is no overlap of subjects (N_0_ = 0) or there are 10,000 shared individuals (N_0_ = 10 000). There is variability in the type I errors and in general, they hover near 0.05, though there appears to be a slight inflation for Q2 and Q3; averaging over all simulation settings (N_1_=15 000; N_2_=50 000; N_0_=0) the average type I errors for Q2 and Q3 are 0.061 and 0.055, respectively.

Figures S3 and S4 display QQ-plots for the overlap models in which the two studies are independent and when they share 10,000 subjects, respectively. Both plots provide support that the type I errors are calibrated well in categories that consist of at least 1% of the variants; the support is not as strong for Q2 and Q3, which are the smallest categories, consisting of fewer than 1% of the variants. The respective inflation factors for Q1 and Q6 are 1.00 and 0.85 in the independent samples setting, and 0.85 and 0.93 in the shared samples setting. Inflation factors calculated from the positive standardised statistics for Q2, Q3, and Q5 are 5.23, 4.52, and 1.00 for independent samples and 5.09, 3.80, and 1.02 for shared samples. Thus, categories that are positive for only 1.40% of the variants (e.g. Q5) are calibrated well for type I error in the overlap model, though there appears to be inflation for the smaller categories (fewer than 1% of variants), Q2 and Q3.

Power and type I error results specific to overlap enrichment settings for Q1 and Q5 are given in Table S7, where study 1 has 15 000 subjects, study 2 has 50 000 individuals and N_o_=0 or N_o_=10 000.

**S.5 Covariate Distributions**

Prior to applying COMET to real data, we considered the distribution of the covariates among variants that are associated (p < 5×10^-6^ as listed by NHGRI and pruned to *r^2^>* 0.1) with fourteen traits/diseases; additional variants associated with body mass index that were not yet listed in NHGRI [accessed 7 October 2015] were also incorporated^12^. Here, we measure association evidence with p-values, rather than converting to ABFs, which requires study-specific information that is not always available (e.g. the effect size and its standard error). Such comparisons are useful as a pre-assessment of the usefulness of the chosen covariates, as to whether or not there is potential for the covariates to differentiate between trait-associated variants for different traits, as well as potential for identifying covariates that may be enriched for shared variants. Observed differences/similarities between phenotypes were assessed by their 95% confidence intervals and their deviation from the proportions under the null hypothesis (estimated from CEU of 1000 Genomes).

For the VEP-annotated covariates listed in Table 1 we examined the proportion of covariate-positive variants (pruned at r^2^>0.1) among variants associated (with p < 5×10^-6^) with fourteen traits: bipolar disorder (BPD), schizophrenia (SCZ), type 1 diabetes (T1D), multiple sclerosis (MS), rheumatoid arthritis (RA), inflammatory bowel disease (IBD), Crohn’s Disease (CD), haematological traits (blood), coronary artery disease (CAD), hypertension/blood pressure (hypten/BP), body mass index/obesity (BMI/obese), type 2 diabetes (T2D), fasting insulin (FI), and fasting glucose (FG). Figure 4 displays the proportion estimates together with 95% confidence intervals, as well as a dashed line indicating the proportion expected under the null hypothesis (as given in Table 1). Among the majority of covariate categories, these confidence intervals suggest enrichment of phenotype-associated variants. Differences/similarities in the covariate distributions among the assortment of traits give support that the selected set of covariates may be of use in determining classes of variants for further searches of predisposing variants (or shared variants) for a specific trait(s).

**S.6 Cell lines, grouped by tissue type, for tissue-specific analyses**

**Liver**

Stellate: hepatic stellate cells

Liver

Hepatocytes: primary hepatocytes

**Liver cancer**

HepG2: hepatocellular carcinoma

Huh7: hepatocellular carcinoma

**Skeletal Muscle**

LHCN-M2: skeletal muscle myoblast

HSMMtube: skeletal muscle myotubes differentiated

SKMC: Skeletal muscle myosatellite cells

Psoas_muscle_OC: Primary frozen psoas muscle tissue

**Cardiac Muscle**

HCM: cardiac myocytes

**Adipose**

Adipocytes

**Pancreas**

HPDE6-E6E7: pancreatic duct immortalized with E6E7 gene of HPV

PanIsletD: dedifferentiated human pancreatic islets

PanIslets: pancreatic islets

References

1. Wakefield J: Bayes factors for genome-wide association studies: comparison with P-values. *Genet Epidemiol* 2009; **33:** 79-86.

2. Marchini J, Howie B, Myers S, McVean G, Donnelly P: A new multipoint method for genome-wide association studies by imputation of genotypes. *Nat Genet* 2007; **39:** 906-913.

3. Wellcome Trust Case Control C: Genome-wide association study of 14,000 cases of seven common diseases and 3,000 shared controls. *Nature* 2007; **447:** 661-678.

4. Asimit JL, Panoutsopoulou K, Wheeler E *et al*: A Bayesian Approach to the Overlap Analysis of Epidemiologically Linked Traits. *Genet Epidemiol* 2015; **39:** 624-634.

5. Purcell S, Neale B, Todd-Brown K *et al*: PLINK: a tool set for whole-genome association and population-based linkage analyses. *Am J Hum Genet* 2007; **81:** 559-575.

6. McLaren W, Pritchard B, Rios D, Chen Y, Flicek P, Cunningham F: Deriving the consequences of genomic variants with the Ensembl API and SNP Effect Predictor. *Bioinformatics* 2010; **26:** 2069-2070.

7. Manning AK, Hivert MF, Scott RA *et al*: A genome-wide approach accounting for body mass index identifies genetic variants influencing fasting glycemic traits and insulin resistance. *Nat Genet* 2012; **44:** 659-669.

8. Saxena R, Hivert MF, Langenberg C *et al*: Genetic variation in GIPR influences the glucose and insulin responses to an oral glucose challenge. *Nat Genet* 2010; **42:** 142-148.

9. Scott RA, Lagou V, Welch RP *et al*: Large-scale association analyses identify new loci influencing glycemic traits and provide insight into the underlying biological pathways. *Nat Genet* 2012; **44:** 991-1005.

**Titles and legends to figures**

**Supplementary Figure S 1: QQ-plots for the covariates in the marginal model for study 1 (N_1_=5 000).** The model is fit to simulated data having p_1_ = 0.04, p_2_ = 0.02, p_12_ = 5×10^-4^ and no covariate categories are set up as enriched.

**Supplementary Figure S 2: QQ-plots for the covariates in the most appropriate overlap model fit to simulated case-control data with shared controls.** The simulations settings have p_1_ = 0.04, p_2_ = 0.02, p_12_ = 5×10^-4^ and no covariate categories are set up as enriched. Study 1 has 5 000 cases, study 2 has 10 000 cases and both studies have 10 000 controls, of which 9 000 are shared.

**Supplementary Figure S 3: QQ-plots for the covariates in the most appropriate overlap model fit to simulated quantitative trait data in which p_1_ = 0.005, p_2_ = 0.005, p_12_ = 2×10^-4^ and no covariate categories are set up as enriched.** The two samples are independent and of sizes N_1_=15 000 and N_2_=50 000.

**Supplementary Figure S 4: QQ-plots for the covariates in the most appropriate overlap model fit to simulated quantitative trait data in which p_1_ = 0.005, p_2_ = 0.005, p_12_ = 2E-04 and no covariate categories are set up as enriched.** The two samples have 10,000 overlapping individuals and are of size N_1_=15,000 and N_2_=50,000.

**Supplementary Figure S 5: Distributions of VEP covariates among common variants (pruned at r^2^>0.1) and significant at 5×10^-6^ for each phenotype.** Each proportion estimate is given together with its 95% confidence interval and the dashed lines represent the expected proportions under the null hypothesis. The covariates correspond to Q1 (upper left), Q3 (upper right), Q5 (lower left), and Q6 (lower right).
