# Supplementary material for: A two-stage inter-rater approach for enrichment testing of variants associated with multiple traits
Source: Eur J Hum Genet. 2016 Dec 21;25(3):341–9. doi: 10.1038/ejhg.2016.171 (PMC5302181; doi:10.1038/ejhg.2016.171)
Supplement: Supplementary Tables [file ejhg2016171x2.docx]

Supplementary Table S 1: Estimates of type I error (including 95% confidence intervals) for the detection as a category positively enriched with overlap signals at coefficient significance level 0.05, for various null settings in the case-control scenario.

|  |  | Type I error (H_1_: ) for each covariate | | | | |
| --- | --- | --- | --- | --- | --- | --- |
| Scenario | Study 1 (cases, controls);  Study 2 (cases, controls) | Q1 | Q2 | Q3 | Q5 | Q6 |
| Null-enrichment;  9,000 shared controls | (5 000, 10 000) (10 000, 10 000) | 0.042 (0.030, 0.054) | 0.063 (0.048, 0.079) | 0.040 (0.028, 0.052) | 0.042 (0.030, 0.054) | 0.033 (0.022, 0.044) |
| Q5-enrichment | (3000, 3000) (5000, 5000) | 0.046 (0.033, 0.059) | 0.058 (0.044, 0.072) | 0.051 (0.037, 0.065) | NA | 0.044 (0.031, 0.057) |
| Q5-enrichment | (5000, 5000)  (10 000, 10 000) | 0.052 (0.038, 0.066) | 0.049 (0.035, 0.062) | 0.053 (0.039, 0.067) | NA | 0.044 (0.031, 0.057) |
| Q5-enrichment | (10 000, 10 000)  (20 000, 20 000) | 0.047 (0.034, 0.060) | 0.048 (0.035, 0.061) | 0.056 (0.042, 0.070) | NA | 0.049 (0.035, 0.062) |
| Q1-enrichment | (3000, 3000) (5000, 5000) | NA | 0.056 (0.042, 0.070) | 0.052 (0.038, 0.066) | 0.048 (0.035, 0.061) | 0.041 (0.029, 0.053) |
| Q1-enrichment | (5000, 5000)  (10 000, 10 000) | NA | 0.065 (0.050, 0.080 | 0.067 (0.052, 0.082) | 0.053 (0.039, 0.067) | 0.035 (0.024, 0.046) |
| Q1-enrichment | (10 000, 10 000) (20 000, 20 000) | NA | 0.060 (0.045, 0.074) | 0.057 (0.043, 0.071) | 0.052 (0.038, 0.066) | 0.045 (0.032, 0.058) |

Supplementary Table S 2: Estimates of the probability of detection as a category positive enriched with overlap signals at coefficient significance level 0.05.

| (A)  N_1_ = 3,000  N_2_ = 5,000 | p_12_’ for Q5  (p_12_’/p_12_ ×100%) | | | | |
| --- | --- | --- | --- | --- | --- |
| Covariate | 7.0×10^-6^  (1.4%) | 2.5×10^-5^  (5%) | 5.0×10^-5^  (10%) | 1.0×10^-4^  (20%) | 2.5×10^-4^  (50%) |
| Q1 | 0.056 | 0.041 | 0.047 | 0.029 | 0.058 |
| Q2 | 0.067 | 0.06 | 0.066 | 0.045 | 0.053 |
| Q3 | 0.057 | 0.055 | 0.045 | 0.053 | 0.046 |
| Q6 | 0.046 | 0.047 | 0.046 | 0.033 | 0.052 |

| (B)  N_1_ = 5,000  N_2_ = 10,000 | p_12_’ for Q5  (p_12_’/p_12_ ×100%) | | | | |
| --- | --- | --- | --- | --- | --- |
| Covariate | 7.0×10^-6^  (1.4%) | 2.5×10^-5^  (5%) | 5.0×10^-5^  (10%) | 1.0×10^-4^  (20%) | 2.5×10^-4^  (50%) |
| Q1 | 0.055 | 0.056 | 0.054 | 0.045 | 0.05 |
| Q2 | 0.056 | 0.043 | 0.059 | 0.046 | 0.043 |
| Q3 | 0.053 | 0.052 | 0.062 | 0.056 | 0.042 |
| Q6 | 0.042 | 0.05 | 0.048 | 0.033 | 0.049 |

| (C)  N_1_ = 10,000  N_2_ = 20,000 | p_12_’ for Q5  (p_12_’/p_12_ ×100%) | | | | |
| --- | --- | --- | --- | --- | --- |
| Covariate | 7.0×10^-6^  (1.4%) | 2.5×10^-5^  (5%) | 5.0×10^-5^  (10%) | 1.0×10^-4^  (20%) | 2.5×10^-4^  (50%) |
| Q1 | 0.046 | 0.049 | 0.045 | 0.051 | 0.047 |
| Q2 | 0.052 | 0.041 | 0.061 | 0.048 | 0.041 |
| Q3 | 0.061 | 0.062 | 0.061 | 0.061 | 0.039 |
| Q6 | 0.052 | 0.041 | 0.062 | 0.046 | 0.047 |

In each of the 1000 simulations, the Q5 category (1.40% of common CEU SNPs LD-pruned at r^2^ > 0.1) was set as enriched for overlap signals. The selected proportion of causal variants in this category is indicated in each column, followed by the proportion among the causal variants p_12_’/p_12_, as a percentage. Studies 1 and 2 are each equal-sized case-control studies of N_1_ each and N_2_ each, respectively; (A) N_1_=3000; N_2_=5000; (B) N_1_=5000; N_2_=10 000; (C) N_1_=10 000; N_2_=20 000.

Supplementary Table S 3: Type I error estimates for the detection as a category positive enriched with overlap signals at coefficient significance level 0.05.

| (A)  N_1_ = 3,000  N_2_ = 5,000 | p_12_’ for Q1  (p_12_’/p_12_ ×100%) | | |
| --- | --- | --- | --- |
| Covariate | 1.0×10^-4^  (20%) | 2.6×10^-4^  (51.5%) | 4.0×10^-4^  (80%) |
| Q2 | 0.062 | 0.051 | 0.055 |
| Q3 | 0.047 | 0.066 | 0.044 |
| Q5 | 0.053 | 0.043 | 0.048 |
| Q6 | 0.047 | 0.046 | 0.029 |

| (B)  N_1_ = 5,000  N_2_ = 10,000 | p_12_’ for Q1  (p_12_’/p_12_ ×100%) | | |
| --- | --- | --- | --- |
| Covariate | 1.0×10^-4^  (20%) | 2.6×10^-4^  (51.5%) | 4.0×10^-4^  (80%) |
| Q2 | 0.065 | 0.064 | 0.068 |
| Q3 | 0.069 | 0.062 | 0.07 |
| Q5 | 0.056 | 0.045 | 0.059 |
| Q6 | 0.028 | 0.039 | 0.038 |

| (C)  N_1_ = 10,000  N_2_ = 20,000 | p_12_’ for Q1  (p_12_’/p_12_ ×100%) | | |
| --- | --- | --- | --- |
| Covariate | 1.0×10^-4^  (20%) | 2.6×10^-4^  (51.5%) | 4.0×10^-4^  (80%) |
| Q2 | 0.06 | 0.060 | 0.061 |
| Q3 | 0.066 | 0.050 | 0.057 |
| Q5 | 0.052 | 0.050 | 0.054 |
| Q6 | 0.053 | 0.042 | 0.042 |

In each of the 1000 simulations, the Q1 category (51.5% of common CEU SNPs LD-pruned at r^2^ > 0.1) was set as enriched for overlap signals. The selected proportion of causal variants in this category is indicated in each column, followed by the proportion among the causal variants p_12_’/p_12_, as a percentage. Studies 1 and 2 are each equal-sized case-control studies of N_1_ each and N_2_ each, respectively; (A) N_1_=3000; N_2_=5000; (B) N_1_=5000; N_2_=10 000; (C) N_1_=10 000; N_2_=20 000.

Supplementary Table S 4: COMET power for detecting Q5 as a category positive-enriched with overlap signals at coefficient significance level 0.05.

|  | p_12_’ for Q5  (p_12_’/p_12_ ×100%) | | | | |
| --- | --- | --- | --- | --- | --- |
| (N_1_, N_2_) | 7.0×10^-6^  (1.4%) | 2.5×10^-5^  (5%) | 5.0×10^-5^  (10%) | 1.0×10^-4^  (20%) | 2.5×10^-4^  (50%) |
| (3,000, 5,000) | **0.045** | 0.324 | 0.778 | 0.811 | 1 |
| (5,000, 10,000) | **0.039** | 0.469 | 0.954 | 0.999 | 1 |
| (10,000, 20,000) | **0.035** | 0.730 | 0.998 | 1 | 1 |

**In each of the 1000 simulations, the Q5 category (1.4% of common CEU SNPs LD-pruned at r^2^ > 0.1) was set to have a certain proportion of shared causal variants. The selected proportion of causal variants in this category p’_12_ is indicated in each column, followed by the proportion among the causal variants p’_12_/p_12_, as a percentage. Studies 1 and 2 are each equal-sized case-control studies of N_1_ each and N_2_ each, respectively. Type I error is denoted by bold font.**

Supplementary Table S 5: COMET power for detecting Q1 as a category positive-enriched or positive/negative enriched with overlap signals at coefficient significance level 0.05.

|  | | p_12_’ for Q1  (p_12_’/p_12_ ×100%) | | |
| --- | --- | --- | --- | --- |
| (N_1_, N_2_) | H_1_ | 1.0×10^-4^  (20%) | 4.0×10^-4^  (80%) | 2.6×10^-4^  (51.5%) |
| (3,000, 5,000) |  | 0.002 | 0.489 | **0.035** |
|  |  | 0.333 | 0.359 | **0.031** |
| (5,000, 10,000) |  | 0 | 0.734 | **0.031** |
|  |  | 0.661 | 0.631 | **0.027** |
| (10,000, 20,000) |  | 0 | 0.919 | **0.024** |
|  |  | 0.885 | 0.852 | **0.016** |

In each of the 1000 simulations, the Q1 category (51.5% of common CEU SNPs LD-pruned at r^2^ > 0.1) was set as enriched for overlap signals. The selected proportion of causal variants in this category p’_12_ is indicated in each column, followed by the proportion among the causal variants p’_12_/p_12_, as a percentage. Studies 1 and 2 are each equal-sized case-control studies of N_1_ each and N_2_ each, respectively. Type I error is denoted by bold font.

Supplementary Table S 6: Estimates of type I error for the detection as a category positively enriched with overlap signals at coefficient significance level 0.05, for quantitative trait simulations.

| N_1_ = 15 000  N_2_ = 50 000 | Type I error (H_1_: ) for covariates in null-enrichment simulations | | | | |
| --- | --- | --- | --- | --- | --- |
| N_o_ | Q1 | Q2 | Q3 | Q5 | Q6 |
| 0 | 0.042 | 0.064 | 0.059 | 0.051 | 0.047 |
| 10 000 | 0.038 | 0.063 | 0.054 | 0.058 | 0.043 |
| N_1_ = N_2_ = 11 000;  N_o_=10 000  (both 91% shared) | 0.047 | 0.066 | 0.058 | 0.035 | 0.056 |
| N_1_ = 10 000;  N_2_ = 15 000  N_o_ = 10 000;  (sample 1 100% shared,  sample 2 67% shared) | 0.036 | 0.059 | 0.064 | 0.060 | 0.051 |
| N_1_ = 10 000;  N_2_ = 12 000  N_o_ = 10 000;  (sample 1 100% shared,  sample 2 83% shared) | 0.058 | 0.063 | 0.063 | 0.055 | 0.054 |
| N_1_ = N_2_ = 10 000;  N_o_ = 10 000  (both 100% shared) | 0.046 | 0.056 | 0.058 | 0.057 | 0.042 |
| N_1_ = 15 000  N_2_ = 50 000 | Average type I error (H_1_: ) for covariates in Q1-enrichment simulations | | | | |
| 0 | NA | 0.053 | 0.055 | 0.045 | 0.041 |
| 10 000 | NA | 0.052 | 0.041 | 0.046 | 0.046 |
| N_1_ = 15 000  N_2_ = 50 000 | Average type I error (H_1_: ) for covariates in Q5-enrichment simulations | | | | |
| 0 | 0.041 | 0.073 | 0.060 | NA | 0.050 |
| 10 000 | 0.041 | 0.061 | 0.059 | NA | 0.045 |

In each of the 1000 simulations, there is no set enrichment of overlap in a pre-specified covariate category. Studies 1 and 2 have N_1_ and N_2_ individuals, respectively, while N_o_ denotes the number of overlapping individuals between the two samples.

Supplementary Table S 7: Estimates of the probability of detection as a category positive-enriched with overlap signals at coefficient significance level 0.05, in quantitative trait simulations.

| N_1_ = 15 000  N_2_ = 50 000 | p_12_’ for Q1  (p_12_’/p_12_ ×100%) | | p_12_’ for Q5  (p_12_’/p_12_ ×100%) | |
| --- | --- | --- | --- | --- |
| **** | 1.0×10^-4^  (50%) | 1.5×10^-4^  (75%) | 2.8×10^-6^  (1.4%) | 2.0×10^-5^  (20%) |
| N_o_ = 0 | **0.018** | 0.35 | **0.039** | 0.71 |
| N_o_ = 10 000 | **0.023** | 0.33 | **0.057** | 0.68 |

In each of the 1000 simulations, either the Q1 or Q5 category was set as enriched for overlap signals. The selected proportion of causal variants in the category is indicated in each column, followed by the proportion among the causal variants p_12_’/p_12_, as a percentage. Studies 1 and 2 respectively contain 15,000 and 50,000 individuals, of which N_o_ are shared between the two studies. Type I error is denoted by bold font.

Supplementary Table S 8: Overlap variants (detected in overlap analysis for each pair of glycaemic traits) that are regulatory in at least one metabolism-involved tissue, as given by RegulomeDB.

|  | Overlap of Fasting Insulin and Fasting Glucose Regulatory Variants | | | | | | | |
| --- | --- | --- | --- | --- | --- | --- | --- | --- |
| SNP | Min  ABF,  Max pvalue | Pancreas | Liver | Cardiac  Muscle | Skeletal  Muscle | Nearest  Gene | Traits associated with SNP or SNP in nearest gene | |
| rs6984305 | 4.10,  3.36  E-07 | * | * | * | * | RP11-115J16.1 | **total, low/high density lipoprotein cholesterol, liver enzyme levels (alkaline phosphatase)** | |
| **rs7903146** | 3.00,  4.81  E-06 | * |  |  |  | TCF7L2 | **2TD, 2G, metabolic syndrome, FG, FI**, BPD | |
| rs13013484 | 2.63,  5.90  E-06 | * | * | * | * | MRPL33 | **total cholesterol, triglycerides**, fasting glucose-related traits (interaction with BMI) | |
| rs7079711 | 1.12,  1.16  E-03 | * |  |  |  | TCF7L2 | T2D, 2G, metabolic syndrome, FG, FI, BPD | |
| rs4736324 | 1.03,  5.88  E-04 | * |  |  |  | LYPD2 | Body fat distribution | |
| **rs10758593** | 0.93,  7.56  E-04 | * |  |  |  | GLIS3 | **T1D,** T2D, thyroid hormone levels, FG | |
| rs2014712 | 0.83,  9.37  E-04 |  | * |  |  | KCNK9 | Adiponectin levels, cholesterol, CAD | |
| rs17126689 | 0.78,  1.99  E-03 | * | * | * | * | AL512791.1 |  | |
| rs4872289 | 0.78,  1.56  E-03 |  |  |  | * | DOCK5 | blood pressure | |
| rs4425665 | 0.75,  3.31  E-03 |  |  |  | * | PRKAG2 | glycogen storage disease of heart, BPD, hemoglobin, red blood cell traits | |
| rs6428891 | 0.70,  4.67  E-03 |  |  |  | * | ATP1A1 | Myocardial infarction | |
|  | Overlap of Fasting Insulin and 2-hour Glucose Regulatory Variants | | | | | | | |
| **rs7903146** | 3.00,  4.81  E-06 | * |  |  |  | TCF7L2 | | **2TD, 2G, metabolic syndrome, FG, FI**, BPD |
| rs17249131 | 0.83,  2.92  E-03 |  | * |  |  | SLC22A23 | | Fasting insulin (interaction), homeostasis model assessment of insulin resistance (interaction), obesity-related traits |
| **rs10758593** | 0.73,  4.16  E-03 | * |  |  |  | GLIS3 | | **T1D,** T2D, thyroid hormone levels, FG |
|  | Overlap of Fasting Glucose and 2-hour Glucose Regulatory Variants | | | | | | | |
| **rs7903146** | 5.96,  2.57  E-08 | * |  |  |  | TCF7L2 | | **2TD, 2G, metabolic syndrome, FG, FI**, BPD |
| rs13266634 | 2.79,  2.00  E-05 | * |  |  |  | SLC30A8 | | **T2D, FG, fasting proinsulin, glycated hemoglobin levles** |
| rs11708067 | 2.48,  6.64  E-05 | * | * |  | * | ADCY5 | | **FG, T2D**, obesity-related, 2G |
| rs598725 | 1.10,  2.20  E-03 |  |  | * | * | RP4-60717.1 | |  |
| rs2881929 | 0.94,  3.65  E-03 |  |  |  | * | CFLAR | | RA |
| rs17383136 | 0.86,  2.59  E-03 |  |  |  | * | RNF220 | | BMI, creatinine |
| **rs10758593** | 0.73,  4.16  E-03 | * |  |  |  | GLIS3 | | **T1D,** T2D, thyroid hormone levels, FG |
| rs10066802 | 0.71,  3.46  E-03 | * | * | * | * | PDE8B | | Thyroid function, thyroid hormone levels |
| rs11220140 | 0.71,  9.12  E-03 | * | * |  | * | STT3A | | SCZ |

An asterisk indicates each tissue that contains the regulatory variant. The nearest gene is also provided, as well as phenotypes that are significant at 5E-06 with the variant (in bold) or with a variant in the nearest gene. SNPs in bold appear in more than one overlap of trait pairs. For reference, the minimum ABF (and maximum p-value) between the two traits is also given, and SNPs are listed in order of evidence strength.

Supplementary Table S 9: Overlap variants for fasting insulin and fasting glucose that are regulatory only in a tissue that is not involved in metabolism, as given by RegulomeDB.

| **SNP** | **Nearest Gene** | Traits associated with SNP or SNP in nearest gene |
| --- | --- | --- |
| rs11576657 | RP5-1185H19.2 |  |
| rs10799891 | RGS4 | SCZ, IBD |
| rs3006928 | AKT3 | Diabetic retinopathy, glucose |
| rs12767087 | C10orf11 | height, hemoglobins |
| rs10771238 | KLRG1 |  |
| rs11174305 | FAM19A2 | cholesterol,LDL, insulin, insulin-resistance, obesity-related traits, triglycerides |
| rs11111272 | IGF1 | insulin-like growth factor I deficiency, FG, FI, height, pulse |
| rs11620369 | GPC6 | BMI,obesity-related traits, visceral fat |
| rs4887904 | ADAMTS18 | BMI |
| rs1118924 | URI1 |  |
| rs2384629 | PPM1G | **triglycerides,** hypertriglyceridemia |
| rs8179219 | GCKR | FG, blood metabolite levels, CAD risk factors, total cholesterol, 2G,FI, lipid metabolism, metabolic traits, triglycerides |
| rs11894322 | NPAS2 |  |
| rs13031932 | ACVR2A | lipoproteins, response to statin therapy |
| rs1467478 | MAFB | total cholesterol |
| rs6070135 | CTCFL | T2D, response to statin therapy, obesity-related traits |
| rs17036328 | PPARG | BMI, lipodystrophy, obesity, FI, T2D |
| rs11720264 | DAG1 |  |
| rs13072457 | RP11-894J14.5 |  |
| rs6894139 | SERBP1P3 |  |
| rs916950 | MEF2C | **height**, BMI, height, SCZ |
| rs697485 | LINC00473 |  |
| rs7004769 | RP11-115J16.1 | **cholesterol, low/high-density lipoprotein cholesterol** |
| rs732839 | RP11-115J16.1 | **total cholesterol,low/high-density lipoprotein cholesterol** |
| rs17120597 | RP11-115J16.1 |  |
| rs1970610 | SGCZ |  |
| rs12552830 | XPA | obesity-related traits, Serum thyroid-stimulating hormone levels |

The nearest gene is also provided, as well as phenotypes that are significant at 5E-06 with the variant (in bold) or with a variant in the nearest gene.
