# Supplementary figures and images for: A two-stage inter-rater approach for enrichment testing of variants associated with multiple traits
Source: Eur J Hum Genet. 2016 Dec 21;25(3):341–9. doi: 10.1038/ejhg.2016.171 (PMC5302181; doi:10.1038/ejhg.2016.171)

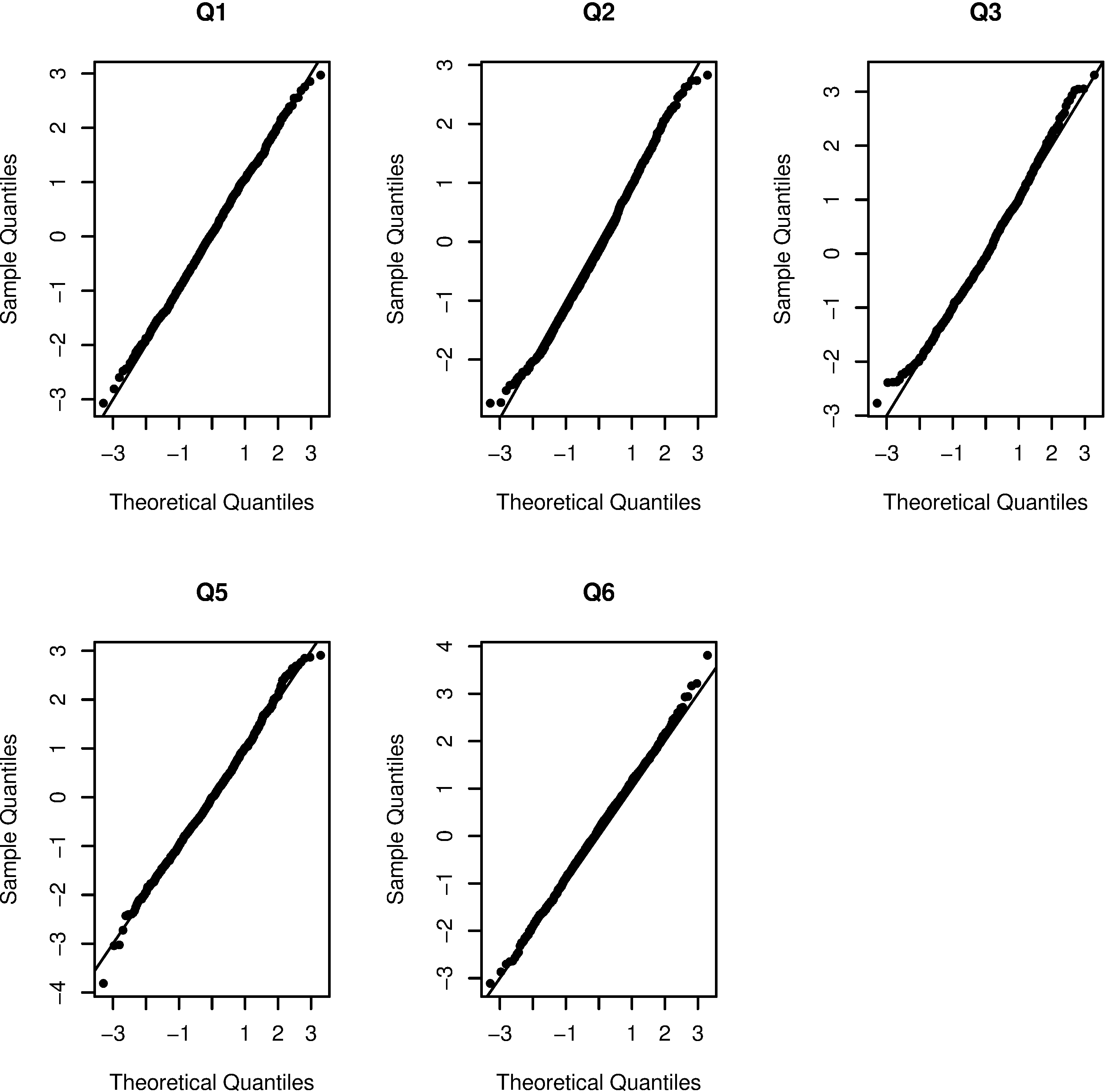

Supplement: Supplementary Figure S1 [file ejhg2016171x3.tif]

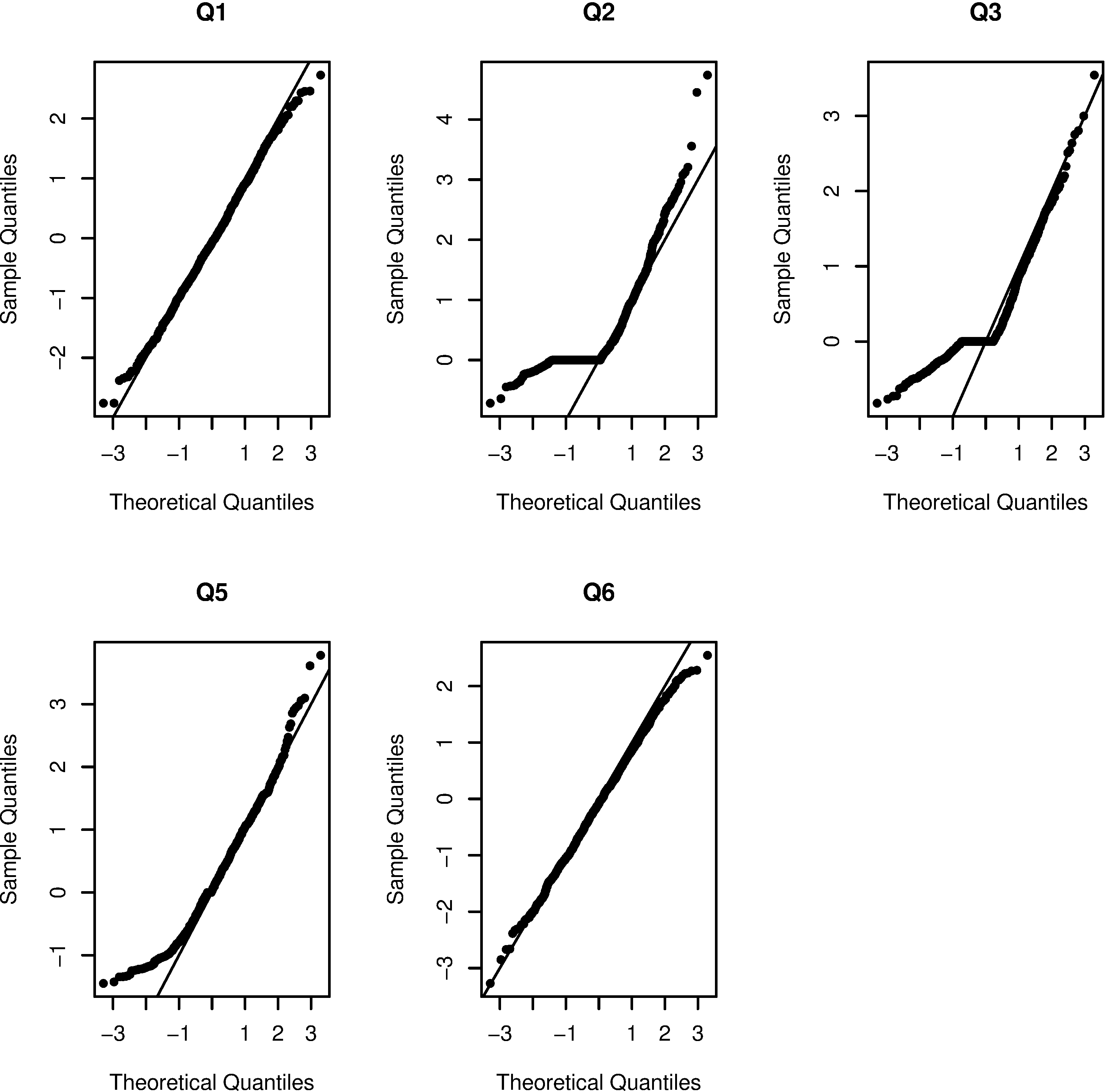

Supplement: Supplementary Figure S2 [file ejhg2016171x4.tif]

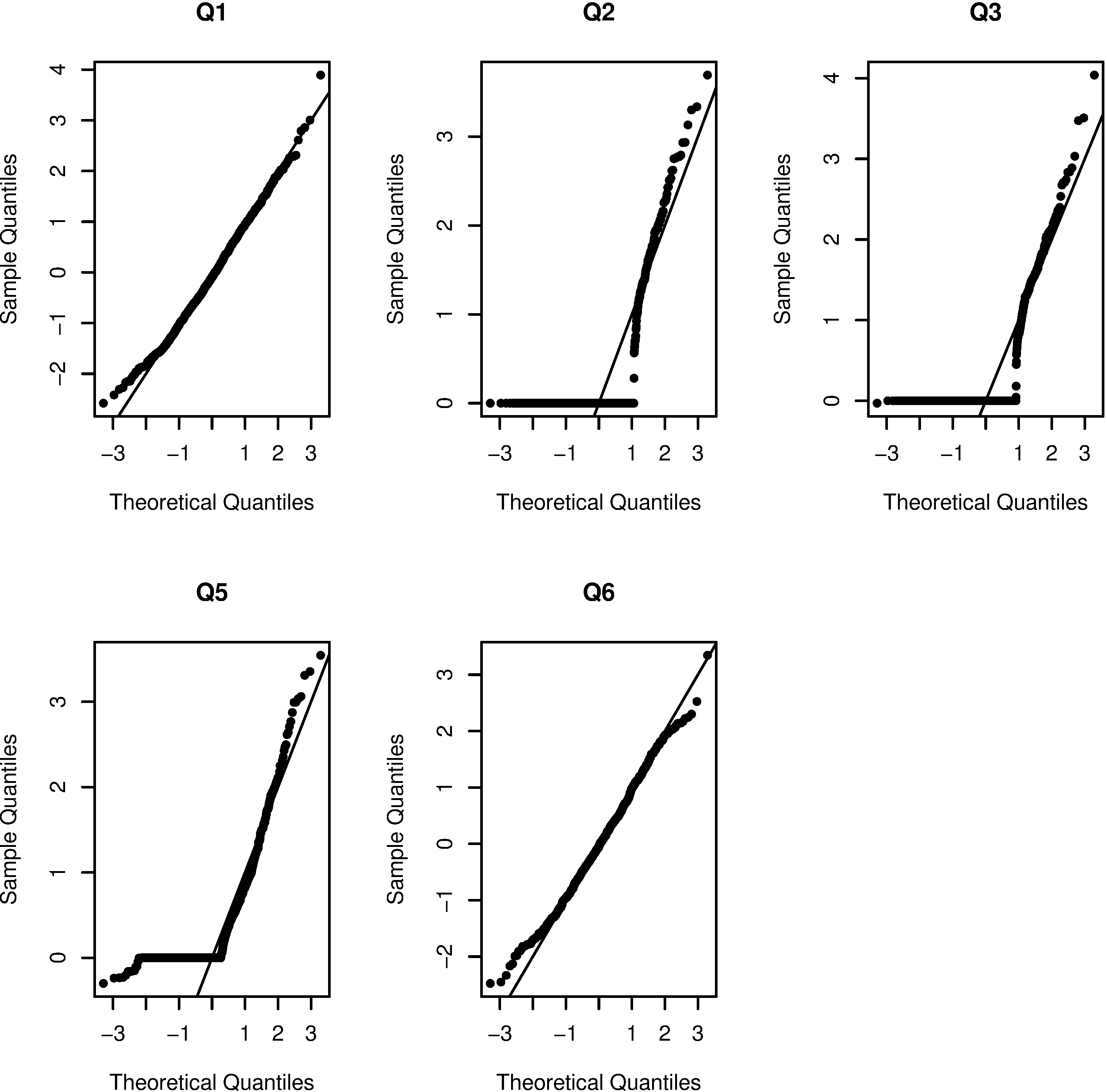

Supplement: Supplementary Figure S3 [file ejhg2016171x5.tif]

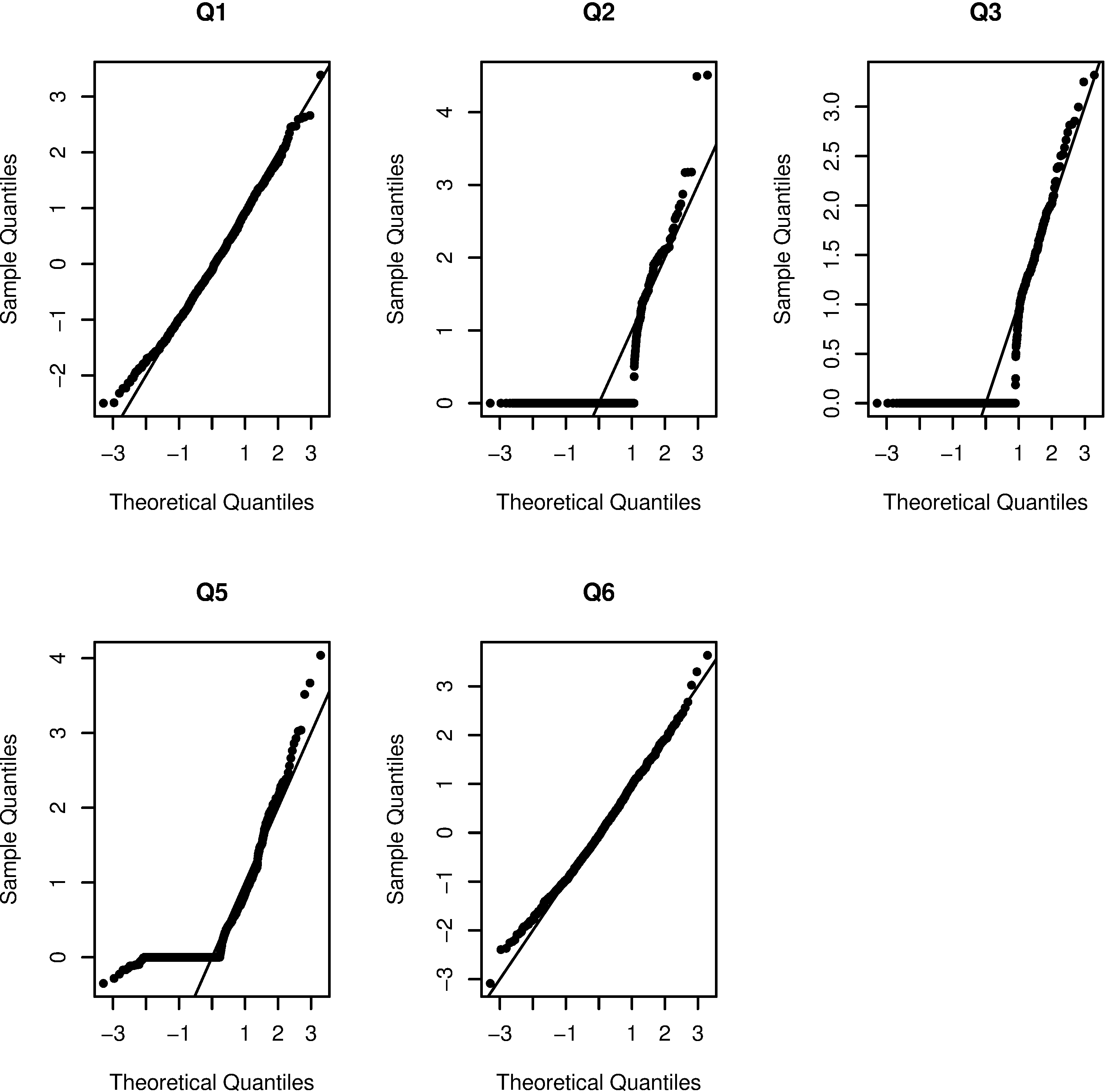

Supplement: Supplementary Figure S4 [file ejhg2016171x6.tif]

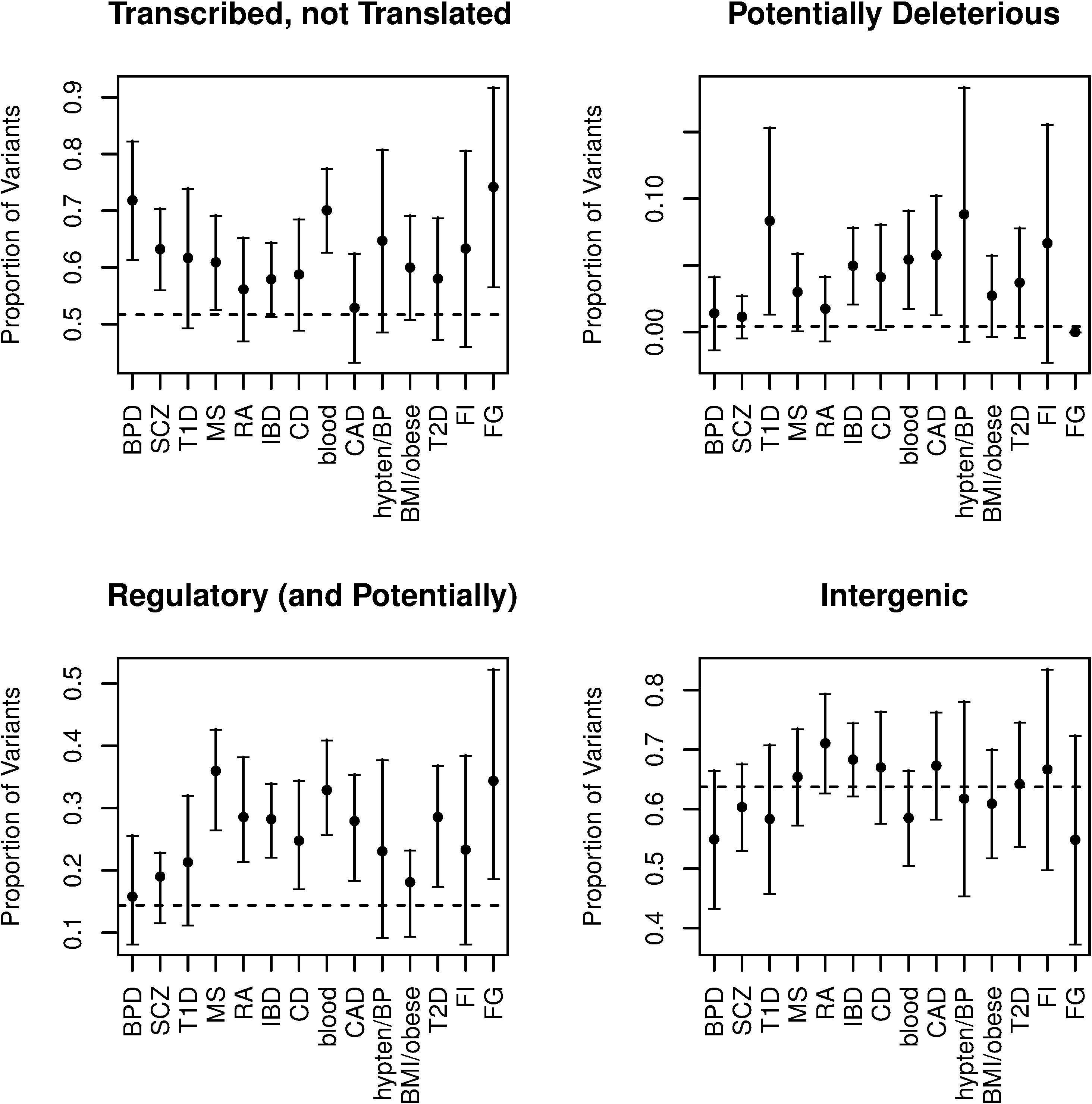

Supplement: Supplementary Figure S5 [file ejhg2016171x7.tif]
